# Comparing efficacies of moxifloxacin, levofloxacin and gatifloxacin in tuberculosis granulomas using a multi-scale systems pharmacology approach

**DOI:** 10.1371/journal.pcbi.1005650

**Published:** 2017-08-17

**Authors:** Elsje Pienaar, Jansy Sarathy, Brendan Prideaux, Jillian Dietzold, Véronique Dartois, Denise E. Kirschner, Jennifer J. Linderman

**Affiliations:** 1 Department of Chemical Engineering, University of Michigan, Ann Arbor, Michigan, United States of America; 2 Department of Microbiology and Immunology, University of Michigan Medical School, Ann Arbor, Michigan, United States of America; 3 Public Health Research Institute and New Jersey Medical School, Rutgers, Newark, New Jersey, United States of America; 4 Department of Medicine, Division of Infectious Disease, New Jersey Medical School, Rutgers University, Newark, New Jersey, United States of America; University of Pennsylvania, UNITED STATES

## Abstract

Granulomas are complex lung lesions that are the hallmark of tuberculosis (TB). Understanding antibiotic dynamics within lung granulomas will be vital to improving and shortening the long course of TB treatment. Three fluoroquinolones (FQs) are commonly prescribed as part of multi-drug resistant TB therapy: moxifloxacin (MXF), levofloxacin (LVX) or gatifloxacin (GFX). To date, insufficient data are available to support selection of one FQ over another, or to show that these drugs are clinically equivalent. To predict the efficacy of MXF, LVX and GFX at a single granuloma level, we integrate computational modeling with experimental datasets into a single mechanistic framework, *GranSim*. *GranSim* is a hybrid agent-based computational model that simulates granuloma formation and function, FQ plasma and tissue pharmacokinetics and pharmacodynamics and is based on extensive *in vitro* and *in vivo* data. We treat *in silico* granulomas with recommended daily doses of each FQ and compare efficacy by multiple metrics: bacterial load, sterilization rates, early bactericidal activity and efficacy under non-compliance and treatment interruption. *GranSim* reproduces *in vivo* plasma pharmacokinetics, spatial and temporal tissue pharmacokinetics and *in vitro* pharmacodynamics of these FQs. We predict that MXF kills intracellular bacteria more quickly than LVX and GFX due in part to a higher cellular accumulation ratio. We also show that all three FQs struggle to sterilize non-replicating bacteria residing in caseum. This is due to modest drug concentrations inside caseum and high inhibitory concentrations for this bacterial subpopulation. MXF and LVX have higher granuloma sterilization rates compared to GFX; and MXF performs better in a simulated non-compliance or treatment interruption scenario. We conclude that MXF has a small but potentially clinically significant advantage over LVX, as well as LVX over GFX. We illustrate how a systems pharmacology approach combining experimental and computational methods can guide antibiotic selection for TB.

## Author summary

Tuberculosis (TB) is caused by infection with the bacterium *Mycobacterium tuberculosis* (Mtb) and kills 1.5 million people each year. TB requires at least 6 months of treatment with up to four drugs, and is characterized by formation of granulomas in patient lungs. Granulomas are spherical collections of host cells and bacteria. Fluoroquinolones (FQs) are a class of drug that could help shorten TB treatment. Three FQs that are used to treat TB are: moxifloxacin (MXF), levofloxacin (LVX) or gatifloxacin (GFX). To date, it is unclear if one FQ is better than the others at treating TB, in part because little is known about how these drugs distribute and work inside the lung granulomas. We use computer simulations of Mtb infection and FQ treatment within granulomas to predict which FQ is better and why. Our computer model is calibrated to multiple experimental data sets. We compare the three FQs by multiple metrics, and predict that MXF is better than LVX and GFX because it kills bacteria more quickly, and it works better when patients miss doses. However, all three FQs are unable to kill a part of the bacterial population living in the center of granulomas. Our results can now inform future experimental studies.

## Introduction

Tuberculosis (TB), caused by *Mycobacterium tuberculosis* (Mtb), is a global public health threat killing 1.5 million people annually [1]. Despite our arsenal of anti-TB antibiotics, effective treatment remains a challenge, requiring at least 6 months of combination therapy with up to four antibiotics. One obstacle to refining TB treatment is complex granuloma structures that develop in patient lungs following infection. Granulomas are dense collections of host immune cells, bacteria and dead host cell debris (caseum); and can be cellular (without caseum), caseous, fibrotic or suppurative (containing neutrophils in the core) [2]. Granulomas isolate Mtb, enhance Mtb replication and provide a potential barrier for antibiotic penetration [3, 4].

Fluoroquinolones (FQs) are a class of antibiotics typically used as second-line agents against multi-drug resistant TB (MDR-TB) [5], or as preventive therapy for MDR-TB contacts [6, 7]. One of three FQs is used in MDR-TB treatment: moxifloxacin (MXF), levofloxacin (LVX) or gatifloxacin (GFX). The choice of one FQ over another is essentially motivated by availability, cost and national guidelines. The WHO recommends use of LVX over MXF, and MXF over GFX [5]. In the absence of comparative clinical trials other than early bactericidal activity [8], there are not sufficient data to declare that treatment with one FQ results in superior clinical outcome. Identifying the best FQ will require careful study of antibiotic dynamics and activity in granulomas.

Recent studies have characterized pharmacokinetic (PK) and pharmacodynamic (PD) metrics of MXF, LVX and GFX (Table 1). The variety of mixed and conflicting data make it unclear whether one FQ is optimal. For example, PK metrics alone indicate: LVX and GFX have higher plasma exposure (area under the concentration curve (AUC)), and MXF has higher concentrations in epithelial lung fluid or alveolar macrophages [9, 10]. Examining PD metrics, GFX has lower MIC against intracellular Mtb, MXF and GFX have equivalent MICs against Mtb grown in liquid culture, MXF has higher bactericidal activity compared to LVX, and MXF and GFX can prevent resistance at lower concentrations than LVX [11–14]. According to clinical metrics, LVX has higher early bactericidal activity (EBA, daily decrease in sputum bacterial burden) (day 0 to 2), all three FQs have equivalent extended EBA (day 2 to 7), and MXF and LVX perform similarly on sputum culture conversion after 3 months, time to sputum culture conversion, and treatment success rate [8, 15–18]. Based on these existing data it is not clear whether one FQ should be preferred for treatment of TB.

**Table 1:** Ranking of MXF, LVX and GFX by different PK, PD or clinical metrics from literature and this work.

Category	Metric	MXF	LVX	GFX	Context	Ref
PK	Plasma exposure relative to effective concentration (AUC/MIC)		*	*	Newly diagnosed TB patients receiving FQs alone prior to standard therapy start.	[9]
PK	Concentration in epithelial lung fluid or alveolar macrophages	*		NS	Five doses in older adults undergoing diagnostic bronchoscopy	[10]
PD	MIC against intracellular Mtb (taking into account intracellular accumulation ratios)			*	Mtb-infected bone marrow derived macrophages from C57Bl/6 mice	This paper
PD	Intracellular antimicrobial activity as measured by CFU after treatment	NS		*	Various concentrations applied to Mtb infected MM6 and A-549 cell lines.	[11]
PD	MIC against Mtb in liquid culture	*		*	Mtb strain H37Rv by turbidimetric assay	This paper
PD	MIC against Mtb in liquid culture	*	NS	*	Mtb strain ATTC 27294 in micro plate Alamar Blue assay	[12]
PD	Bactericidal activity, measured as % inhibition of bacterial growth	*		NS	Clinical strains in liquid culture	[13]
PD	Concentration required to prevent resistant mutants from being recovered	*		*	Clinical Mtb strains grown on Middlebrook 7H11 plates	[14]
Clinical	Early bactericidal activity as measured by sputum CFU decline in day 0 to 2 of therapy		*		Newly diagnosed TB patients receiving 7 days of FQs alone, prior to standard therapy start.	[8]
Clinical	Early bactericidal activity as measured by sputum CFU decline in day 2 to 7 of therapy	*	*	*	Newly diagnosed TB patients receiving 7 days of FQs alone, prior to standard therapy start.	[8]
Clinical	Sputum culture conversion after 3 months	*	*	NS	MDR patients, FQs given in addition to background regimens	[15]
Clinical	Time to sputum culture conversion, treatment success rate.	*	*	NS	Retrospective study of MDR patients treated with regimens containing MXF or LVX.	[16]
Clinical	Treatment success (cure)	*	*	NS	Retrospective study in MDR patients treated with regimens containing MXF or LVX	[17]
Clinical	Decline in serial sputum bacterial load	*	NS	*	Phase II trial replacing MXF or GFX for Ethambutol in standard treatment	[18]

* means FQ is best based on each metric, if more than one FQ is marked, they were considered equivalent.

NS: not studied. MIC: minimum inhibitory concentration.

AUC: Area under the concentration curve.

The ability of an antibiotic to successfully treat TB depends on complex interactions along its path from dose, to plasma, to granuloma to bacterium [19]. Antibiotic concentrations in the blood determine how much antibiotic is available for distribution into granulomas. Antibiotics diffuse from blood vessels into lung tissue and granulomas, where spatial distribution is affected by uptake into host-cells, binding to caseum, and location of functional blood vessels. Once an antibiotic reaches bacteria, it must penetrate the bacterial cell wall and reach the molecular target in sufficient concentration to kill. Complicating things further is inter-individual host variability in plasma pharmacokinetics and lung pathology (lesion type) that must be considered when predicting antibiotic efficacy in TB.

Both experimental and computational studies can be useful to identify antibiotic regimens that will effectively treat TB. Experiments can quantify antibiotic concentrations, spatial distributions, *in vitro* activity and *in vivo* efficacy in animal models or as part of background regimens in humans (Table 1). Computational approaches can combine datasets from multiple experimental systems, interpolate between experimental data points, and screen large numbers of treatment regimens more time- and cost-effectively [20–22]. Here we take a systems pharmacology approach, integrating state-of-the-art experimental and computational methods to predict FQ efficacy and compare FQs.

We present a spatio-temporal computational model of granuloma formation, function and treatment that is calibrated to an exhaustive experimental dataset. Data include FQ dynamics in blood plasma, spatial and temporal distribution in granulomas, and activity *in vitro*. Our hybrid computational model, *GranSim*, tracks events at multiple spatial scales (molecular, cellular and tissue) and time scales (seconds to months). Our systems pharmacology approach provides a unique format for predicting, at a single granuloma level, the potential effects of these three different FQs.

## Results

### Computational model integrates experimental fluoroquinolone dynamics

**Fig 1:**
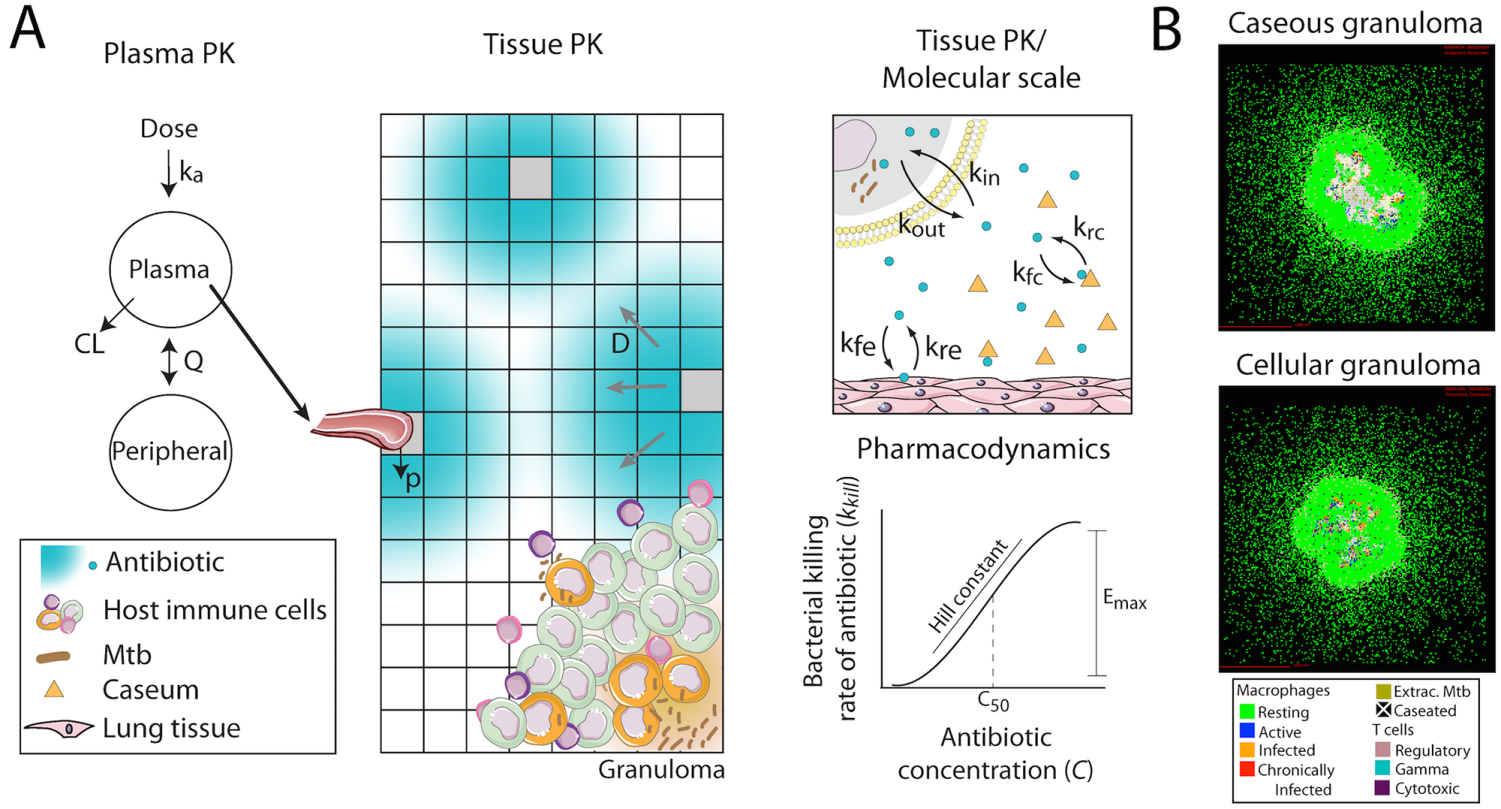
Computational model structure and simulated granulomas. (A) The model tracks plasma PK dynamics using a two-compartment model. The plasma PK model is linked to an agent-based model (ABM) representing spatial and temporal granuloma formation as well as tissue PK, i.e. antibiotic diffusion in the lung tissue and penetration into the granuloma. The model also tracks molecular level antibiotic dynamics such as cell uptake, and caseum binding. Finally the model calculates the antibacterial activity of antibiotics at specific locations in the granuloma using an E_max_ model based on local antibiotic concentration. Parameter definitions are in Table 3. (B) Emergent behavior of the model system is the formation of *in silico* granulomas that can represent the spectrum of granulomas observed *in vivo*, e.g. caseous and cellular granulomas shown. Art adapted from Servier Medical Art (http://servier.com/Powerpoint-image-bank) provided under a Creative Commons Attribution 3.0 Unported License.

To predict and compare FQ efficacy in granulomas we use our mechanistic computational model, *GranSim* (Fig 1) [23–26]. *GranSim* is a spatio-temporal model of granuloma formation and function that incorporates macrophage and T cell recruitment, migration and interaction; secretion and diffusion of chemokines and cytokines; Mtb growth and phagocytosis; and caseation. In the context of these *in silico* granulomas, *GranSim* simulates antibiotic plasma PK, tissue PK and PD [20–22]. *GranSim* is implemented as in [20–22] with three updates made in this work: 1) inclusion of fluoroquinolone dynamics (previous versions include only isoniazid and rifampin); 2) dynamic representation of cellular uptake of antibiotics (previous versions assume pseudo-steady state); and 3) antibiotic binding to caseum and normal lung tissue (previous versions approximate binding by using effective diffusivity parameters). These changes were necessary for model calibration to experimental FQ data.

**Fig 2:**
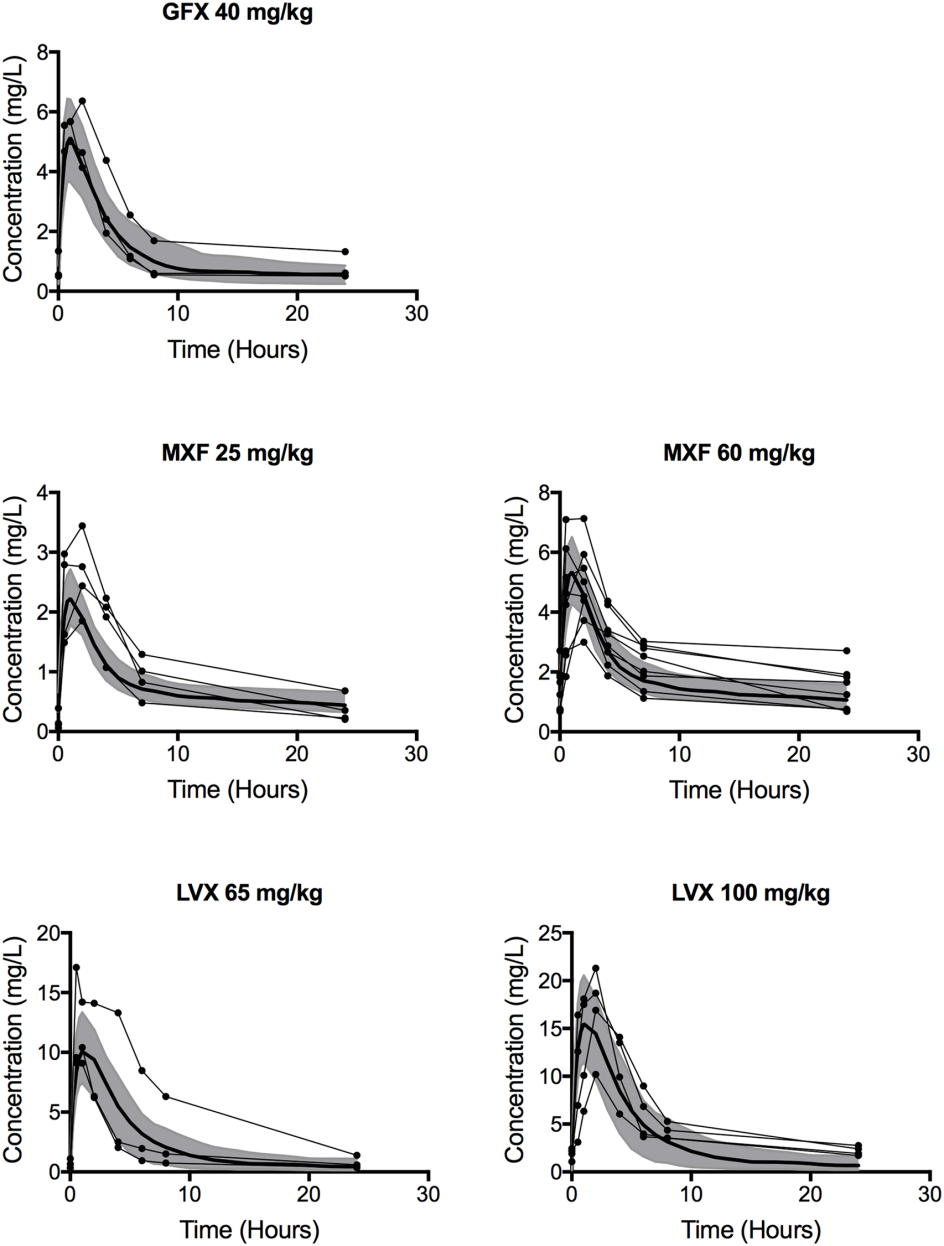
Comparison of plasma PK in rabbits and simulations. A two-compartment plasma PK model describes plasma dynamics in rabbits. To show variation in simulation outcomes we plot both baseline simulations (dark lines) and standard deviations (shaded) for 100 simulations. To show variation in rabbit data we plot individual measurements (connected circles) for between 3 and 7 rabbits.

**Fig 3:**
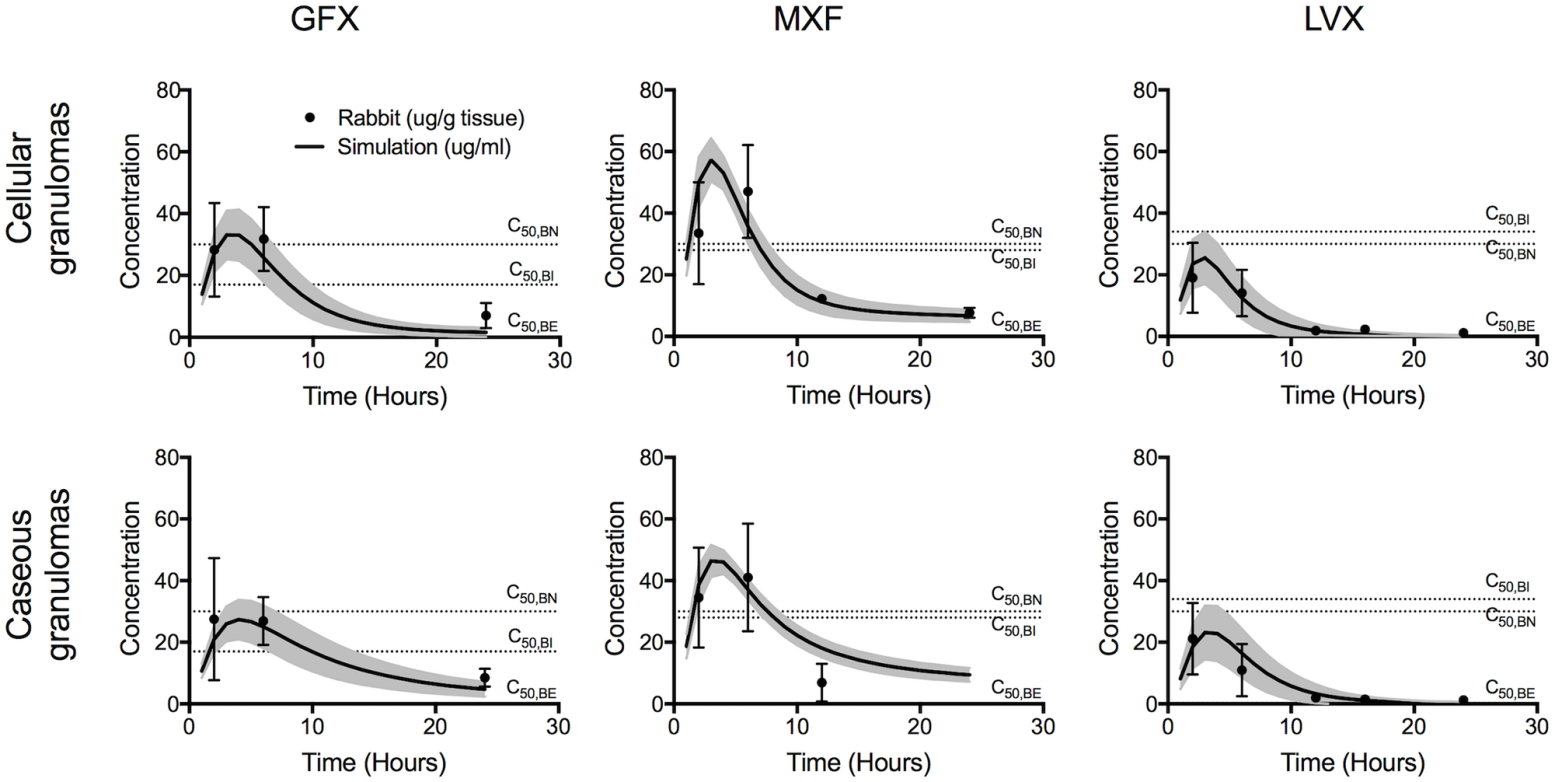
Comparison of average FQ concentrations in simulated granulomas (solid lines) recapitulate LCMS measurements in rabbit granulomas (data points). Lines and data points show means and standard deviations for 100 *in silico* granulomas, and between 1 and 67 rabbit granulomas. Horizontal dotted lines show C_50_ values for intracellular (C_50,BI_), extracellular replicating (C_50,BE_) and extracellular non-replicating bacteria (C_50,BN_). Though not used for model calibration, dynamics in uninvolved lung are also in agreement between simulations and rabbit data (Figure in S2 Fig).

**Fig 4:**
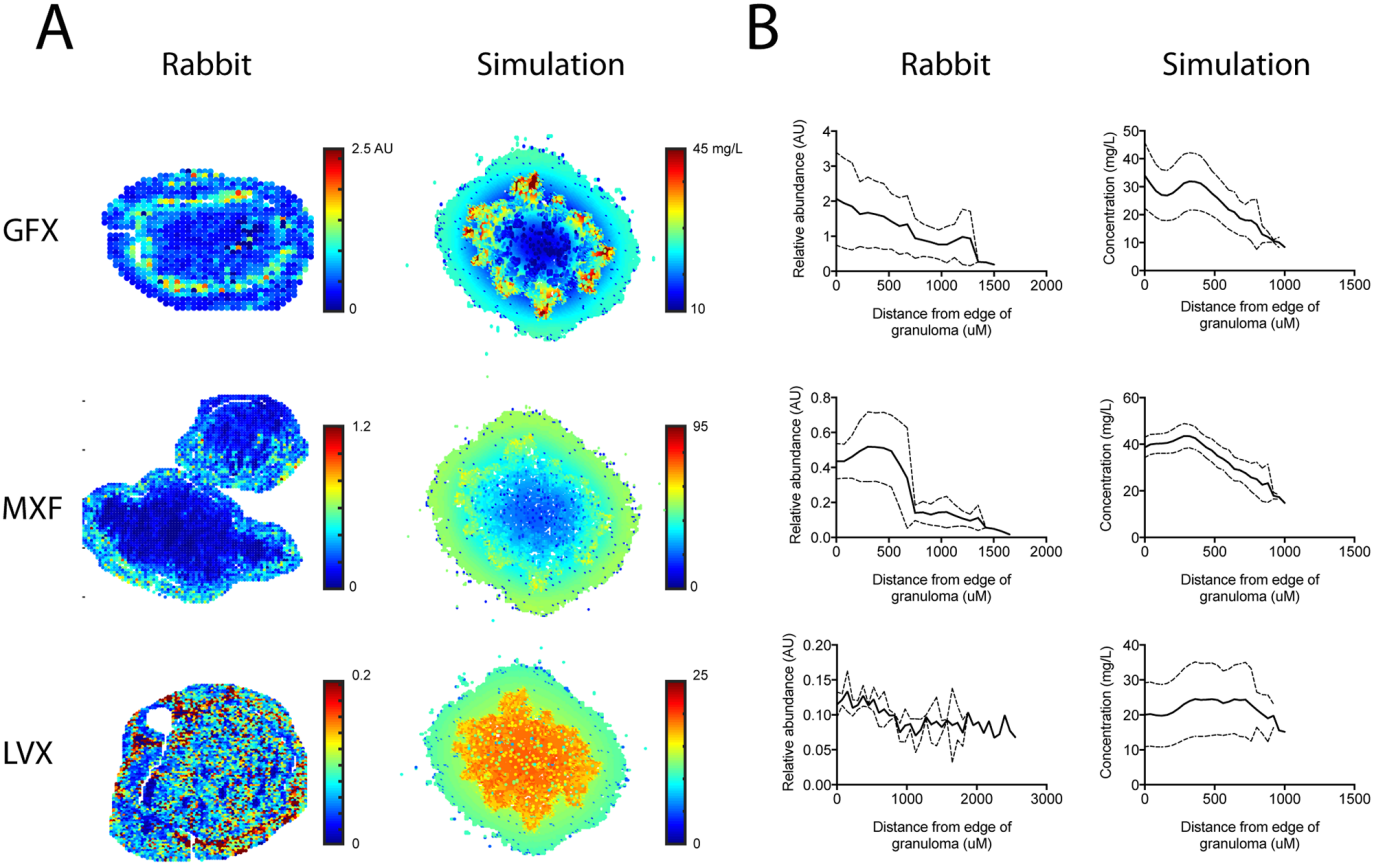
Spatial distributions of FQs in simulated granulomas recapitulate MALDI-MS imaging in rabbit granulomas. (A) Representative granulomas from rabbits (left) and simulations (right) showing different spatial distribution of GFX, MXF and LVX at 6 hours post dose. Simulations capture the qualitative differences between the three FQs. A quantitative comparison between our simulations and MALDI-MSI is not possible due to the semi-quantitative nature of the MALDI-MSI data. (B) Different distributions between GFX, MXF and LVX are consistent across all granulomas studied. Figures show average MALDI-MS abundance in rabbit granulomas (left) and concentrations in simulated granulomas (right) plotted as a function of distance from the edge of the granuloma (in μm). Solid lines show mean and dashed lines show standard deviation for 100 simulated granulomas and between 3 and 7 rabbit granulomas.

We estimate *GranSim* parameters by calibrating to plasma PK, tissue PK, and PD data from *in vitro* and rabbit studies performed in this work, and from human studies described in the literature (Table 2). The plasma PK model within *GranSim* reproduces rabbit plasma concentrations of FQs (Fig 2). *GranSim* captures temporal concentration measurements in homogenized cellular and caseous rabbit granulomas (Fig 3), as well as qualitative differences in the spatial distribution of the FQs (Fig 4). PD parameters reproduce *in vitro* dose response curves specific to different bacterial subpopulations (intracellular, extracellular replicating or extracellular non-replicating) (Figure in S1 Fig). Parameters used for simulations are listed in Table 3.

**Table 2:** Summary of experimental dataset types and where they are integrated into the computational model.

Computational model component	Experimental datasets used for calibration
Plasma PK	Plasma pharmacokinetic studies in rabbits (this work) and humans [9]
Tissue PK	Physicochemical data [27] Drug uptake in human cells Caseum binding Temporal granuloma pharmacokinetic studies (LC/MS-MS) Spatial granuloma pharmacokinetic studies (MALDI-MSI)
Intracellular PD	Intracellular dose response curves Drug uptake in human cells
Extracellular PD	Dose response curves in liquid culture

Data are from this work, unless otherwise specified.

**Table 3:** Model parameters by computational model component.

Parameter	Units	MXF	GFX	LVX
**Rabbit Plasma PK parameters** ^(^^1^^,^^3^^)^				
Absorption rate constant (*k*_*a*_)	h^-1^	0.9, 57	0.46, 77	1.25, 50
Intercompartmental clearance rate constant (*Q*)	L/h/kg	2.86, 12	1.15, 44	0.5, 174
Plasma volume of distribution (*V*_*p*_)	L/kg	5.35, 9	1.27, 39	3.48, 10
Peripheral volume of distribution (*V*_*pe*_)	L/kg	80.8, 0.9	90, 1.5	130, 2
Plasma clearance rate constant (*CL*)	L/h/kg	1.3, 27	1, 61	0.82, 96
**Human Plasma PK parameters** ^(^^2^^,^^3^^)^				
Absorption rate constant (*k*_*a*_)	h^-1^	2.27, 0.21	1.29, 0.4	6.24, 1.4
Intercompartmental clearance rate constant (*Q*)	L/h/kg	9.44, 0.67	31.88, 0.76	0.8, 0.82
Plasma volume of distribution (*V*_*p*_)	L/kg	0.37, 0.29	0.93, 0.67	0.8, 0.33
Peripheral volume of distribution (*V*_*pe*_)	L/kg	0.67, 0.19	0.31, 0.37	0.46, 0.5
Plasma clearance rate constant (*CL*)	L/h/kg	0.12, 0.16	0.17, 0.14	0.12, 0.2
**Lung tissue PK parameters** ^(^^4^^)^				
Effective diffusivity (*D*)	cm^2^/s	1.4x10^-7^	1.2x10^-7^	1.3x10^-6^
Cellular accumulation ratio ^(^^2^^)^ (*a*)	-	7.00	2.75	3.81
Vascular permeability (*p*)	cm/s	3.0x10^-6^	1.3x10^-6^	2.0x10^-6^
Permeability coefficient (*PC*)	-	4.87	3.4	1.56
Caseum unbound fraction (*f*_*u*_)	-	0.26	0.23	0.36
Caseum binding rate constant (*k*_*fc*_)	cu^-1^s^-1^	0.003	0.006	0.007
Epithelium binding association constant (*K*_*a*_)	-	0.01	0.01	0.02
Epithelium binding rate constant (*k*_*fe*_)	s^-1^	0.005	0.01	0.01
Cellular exit rate constant (*k*_*out*_)	s^-1^	0.11	0.12	0.16
**PD parameters** ^(^^5^^)^				
Max activity extracellular (*E*_*max*,*BE*_)	s^-1^	0.003	0.004	0.004
Max activity intracellular (*E*_*max*,*BI*_)	s^-1^	0.010	0.008	0.010
C50 for extracellular replicating Mtb (C_*50*,*BE*_)	mg/L	0.06	0.06	0.23
C50 for extracellular non-replicating Mtb (*C*_*50*,*BN*_) ^(^^6^^)^	mg/L	30.00	30.00	30.00
C50 for intracellular Mtb (*C*_*50*,*BI*_)	mg/L	28.36	17.03	34.31
Hill constant for intracellular Mtb (*H*_*BI*_)	-	1.18	1.32	1.61
Hill constant for extracellular replicating Mtb (*H*_*BE*_)	-	4.99	3.95	5.02
Hill constant for extracellular non-replicating Mtb (*H*_*BN*_)	-	4.99	3.95	5.02

^(1)^ Estimated here based on rabbit plasma PK data collected in this work.

^(2)^ Estimated here based on data from [9].

^(3)^ Values shown are mean and % coefficient of variation determined by non-linear mixed effects modeling.

^(4)^ Estimated here based on rabbit tissue PK data collected in this work.

^(5)^ Estimated here based on *in vitro* data collected in this work.

^(6)^ Values obtained from [27]

Interesting comparisons between the FQs emerge from the calibrated model. Plasma PK suggest higher peak concentrations of LVX and GFX (Fig 2), and faster inter-compartmental clearance for MXF (Table 3). Contrary to plasma PK, data from homogenized granulomas reveal higher MXF peak concentrations compared to GFX and LVX (Fig 3). This finding that MXF peak concentrations are lower in plasma and higher in granulomas compared to GFX and LVX highlights the need for more detailed PK studies in granulomas. Spatial distribution of FQs in rabbit and simulated granulomas reveal: poor penetration of GFX and MXF into caseum compared to LVX, GFX accumulation in cellular areas immediately surrounding caseum, and more evenly distributed MXF accumulation in cellular areas of granulomas (Fig 4). These spatial data suggest that average concentrations in homogenized granulomas might not represent antibiotic dynamics at specific locations where bacteria reside, e.g. caseum. PD parameter values show that MXF and GFX have similar *C*_*50*_ values (concentration where 50% of maximum activity is achieved), while LVX has higher *C*_*50*_ values against both intracellular and extracellular Mtb.

Model parameters also provide insight into the mechanisms behind FQ spatial distribution and function. Tissue PK parameter values indicate that: higher penetration of LVX into caseum is due to higher effective diffusivity in granulomas and slightly lower binding to caseum; accumulation of MXF in cellular areas is due to higher uptake into host cells; and GFX accumulation around caseum is due to slightly faster binding to caseum compared to MXF. PD parameter values indicate that MXF, LVX and GFX all have steep dose response curves, suggesting that these drugs would have very little effect at sub-*C*_*50*_ concentrations.

Integration of multiple datasets ensures that our computational model captures spatial and temporal *in vivo* dynamics, while being consistent with *in vitro* and literature observations. Predictions for FQ efficacy in granulomas are now possible. For example, is better penetration of LVX into the caseum able to overcome its higher *C*_*50*_ values (i.e. are spatial granuloma PK differences relevant in the context of PD differences)? Is the higher concentration of MXF in granulomas able to overcome its poor relative penetration into caseum (i.e. are spatial granuloma PK differences relevant in the context of temporal PK differences)? We use our systems pharmacology model to address these questions.

### Spatial fluoroquinolone distribution in rabbit granulomas is representative of distribution in humans

Before predicting FQ efficacy we explore how human vs. rabbit plasma PK affects the distribution of FQs in granulomas. There are notable differences in FQ plasma PK between rabbits and humans. To predict if human plasma PK would affect the observed spatial distribution of FQs within granulomas, we simulate treatment using human plasma PK parameters that we fit to existing data [9]. Parameter differences between rabbit and human data suggest faster absorption and slower clearance of all three FQs in humans compared to rabbits (Table 3). Spatial distributions in simulated granulomas are similar when using human or rabbit plasma PK parameters (Figure in S3 Fig). Temporal differences in granulomas reflect differences in plasma PK between rabbits and humans, most notably more antibiotic accumulation within granulomas at 24 hours post dose with human plasma PK compared to rabbit plasma PK. This is a result of slower plasma clearance in humans (Table 3), resulting in slower movement of FQs out of the lung tissue into blood following peak concentrations. These results indicate that the qualitative spatial distribution of FQs is relatively insensitive to plasma PK. Taken together, our simulations suggests that the rabbit model provides an accurate representation of FQ spatial distribution within human granulomas if the granulomas are similar.

### MXF has small efficacy advantages over LVX, and LVX over GFX

**Fig 5:**
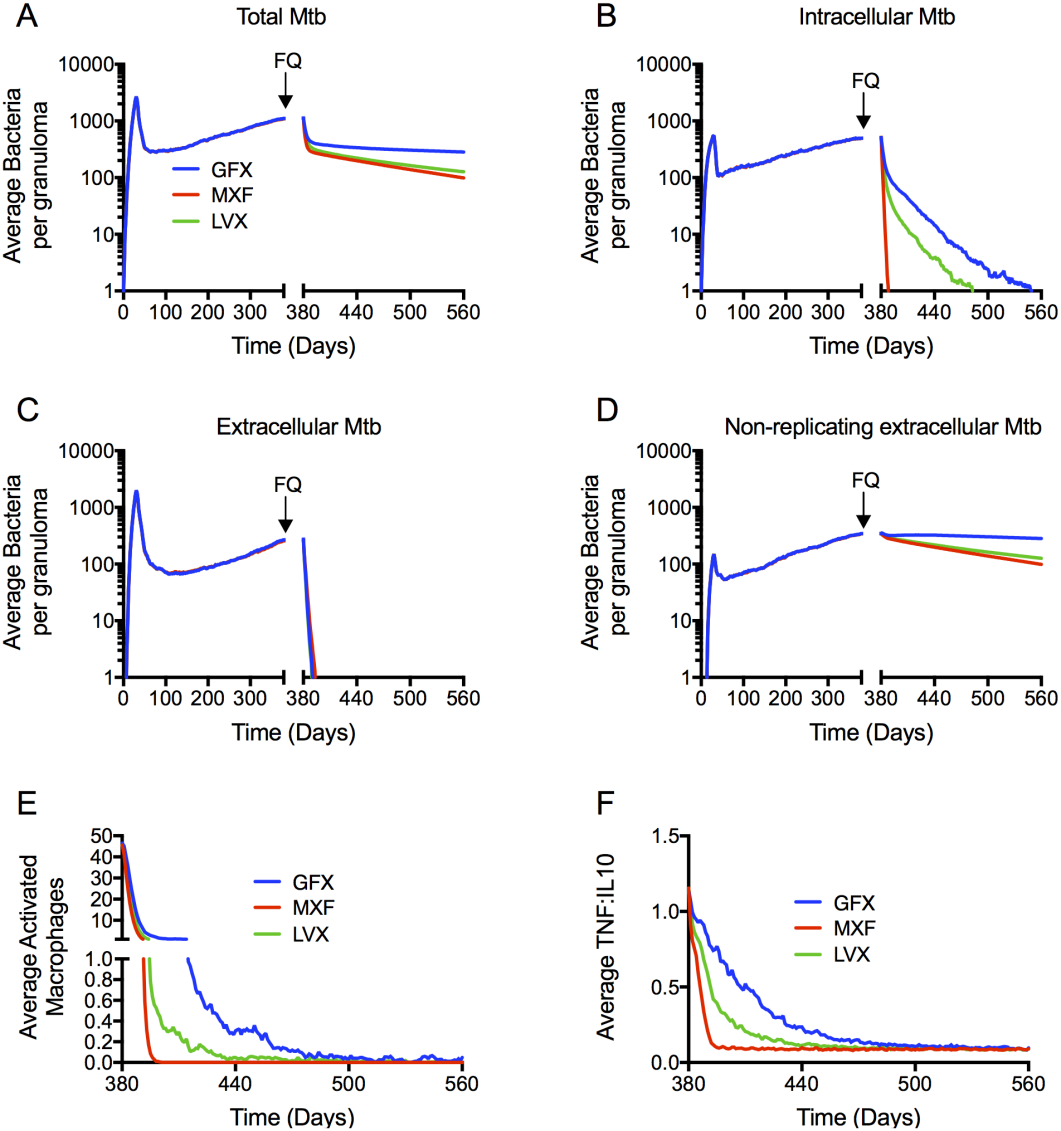
Simulated bacterial CFU (A-D) and host immune dynamics (E-F) during treatment are similar between MXF and LVX with GFX killing fewer bacteria. Dynamics in the total bacterial load (A) are dominated by the non-replicating Mtb population residing the caseum (D), which is not being cleared by any of the FQs. MXF clears the intracellular bacterial population (B) more quickly than LVX and GFX, and all three FQs clear the extracellular bacterial population within days (C). Metrics of inflammation (number of activated macrophages (E), and TNF-α:IL-10 ratio (F)) decline more quickly during MXF treatment compared to LVX and GFX. Lines show means of 210 *in silico* granulomas, with infection starting at day 0, and daily FQ treatment starting at day 380 (arrows) for 6 months (ending at day 560).

To compare FQ efficacy, we simulate 6 months of daily therapy with each FQ in a collection of 210 *in silico* granulomas, starting at day 380 post-infection, using human plasma PK parameters. We use the following metrics to quantify FQ efficacy: bacterial load per granuloma during and after treatment, percentage of granulomas sterilized and time to sterilization. We also characterize FQ treatment in terms of immune responses within granulomas. Bacterial load per granuloma during and after treatment is similar for MXF and LVX (Fig 5A), with higher bacterial load following GFX treatment. For all three FQs, treatment responses comprise a sharp initial decline followed by a slower decline. This biphasic response is widely observed during TB therapy [28]. Evaluating the response of specific bacterial subpopulations to treatment reveals that MXF sterilizes the intracellular bacterial subpopulation more quickly than LVX and GFX (Fig 5B). The extracellular replicating subpopulation is effectively sterilized (falling below an average of 1 bacterium per granuloma) within 10 to 13 days by all three FQs (Fig 5C). The non-replicating subpopulation (residing in the caseum) shows a very slow decline for all three FQs (Fig 5D), and is responsible for the second phase in the biphasic kill curve in Fig 5A.

Besides FQ effects on bacterial load within granulomas, we can also track immunological changes within granulomas during treatment. Upon infection, macrophages in the lung are activated in response to inflammatory cytokines (TNF-α, IFN-γ) and/or the presence of bacteria [29–31]. Furthermore, computational and non-human primate studies have shown that a balance between concentrations of inflammatory cytokines (e.g. TNF-α) and anti-inflammatory cytokines (e.g. IL-10) is an important determinant in controlling bacterial growth in granulomas while limiting tissue damage [23, 32]. The number of activated macrophages and the ratio of TNF-α concentration to IL-10 concentration in our simulation is therefore used as two metrics of inflammation. The inflammation metrics reflect the predicted bacterial differences between the FQs. MXF sterilizes the intracellular population more quickly than LVX and GFX, thereby eliminating infected macrophages, which are important drivers of host inflammation. As a result, numbers of activated macrophages and ratio of TNF-α concentration to IL-10 concentration both decline more quickly during MXF treatment compared to LVX and GFX (Fig 5E and 5F). Other metrics of inflammation (e.g. number of activated cytotoxic or IFNγ-producing T cells) show little differences between FQs. These results suggest that bacterial killing by immune mechanisms continue to play a role during GFX and LVX treatment, and are less prominent during MXF treatment.

**Fig 6:**
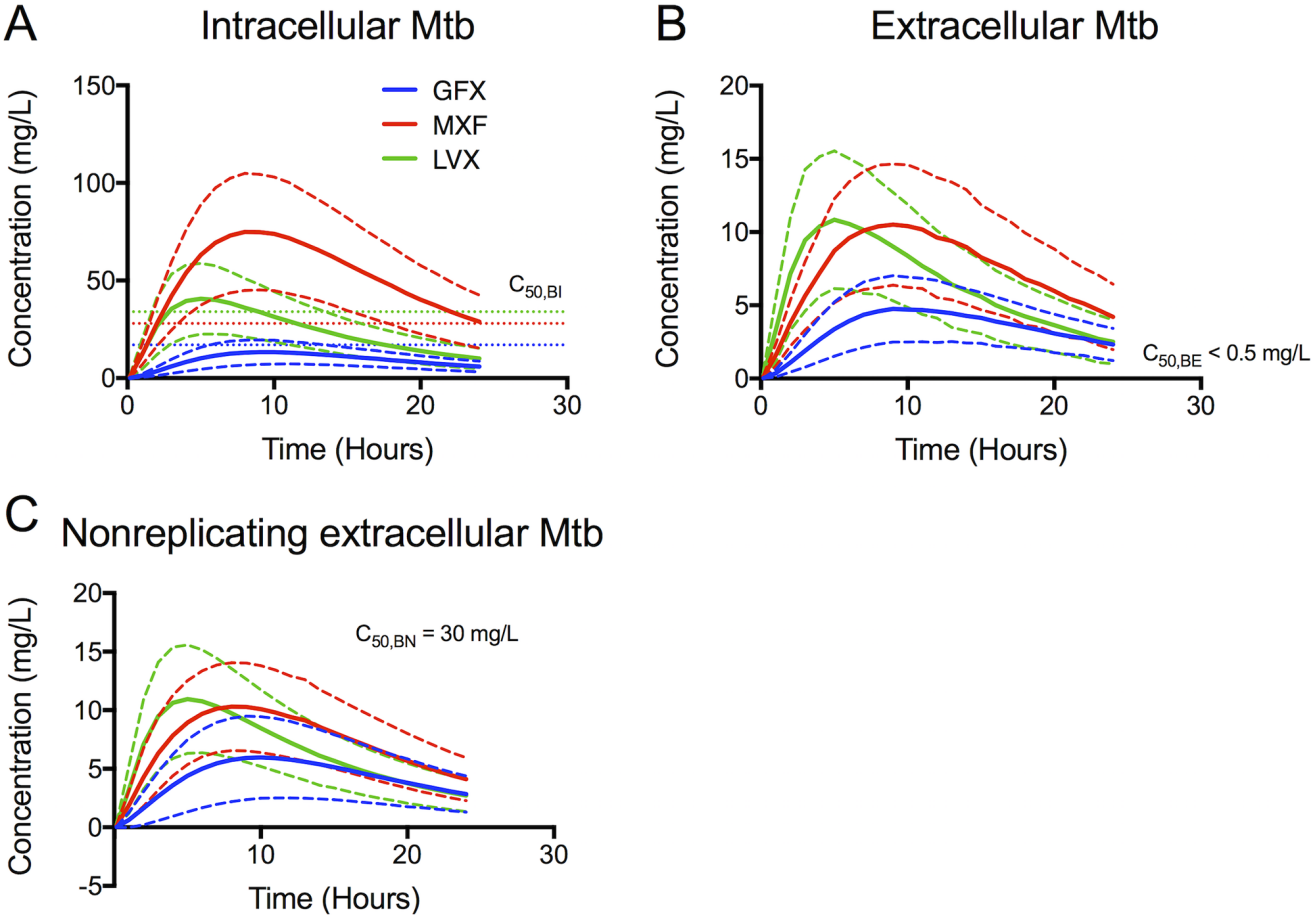
Simulated average free FQ concentrations that each bacterial subpopulation (intracellular (A), extracellular (B) and nonreplicating (C)) is exposed to during one dosing period. Solid lines show means and dashed lines show standard deviations for 210 *in silico* granulomas. Horizontal dotted lines show C_50_ values for intracellular (C_50,BI_), extracellular replicating (C_50,BE_) and extracellular non-replicating bacteria (C_50,BN_) for each FQ.

The FQ concentration experienced by each of the bacterial subpopulations (Fig 6) reveals the cause for the observed bacterial load dynamics. Intracellular bacteria are exposed to concentrations above *C*_*50*,*BI*_ for more than half of the dosing period for MXF, but not LVX or GFX (Fig 6A). Extracellular replicating bacteria are exposed to FQ concentrations well in excess of their effective concentrations throughout most of the dosing period (Fig 6B). We predict that non-replicating subpopulations see concentrations ~3-fold lower than their effective concentrations (Fig 6C).

**Fig 7:**
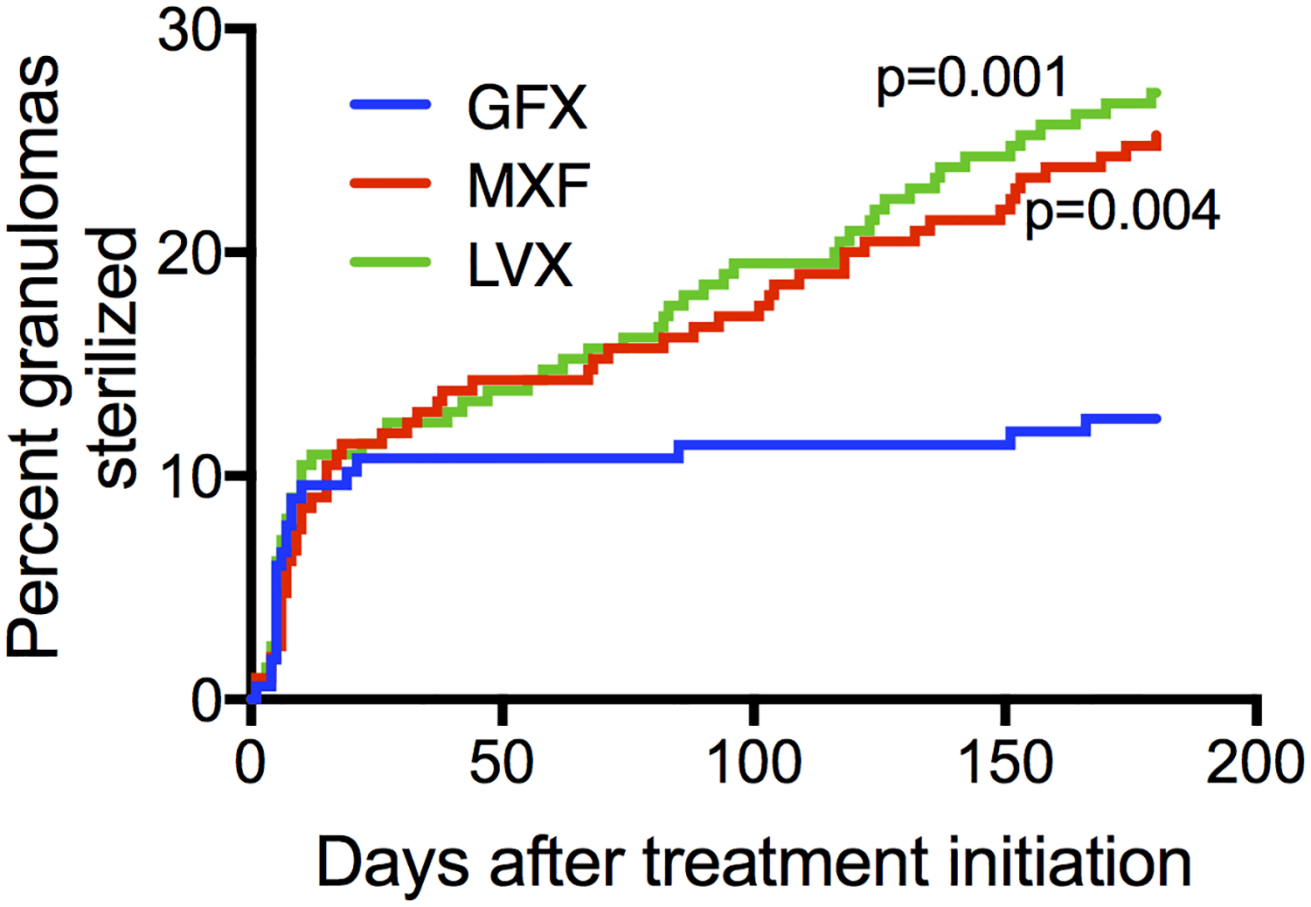
Simulations show that MXF and LVX sterilize more granulomas than GFX. Kaplan-Meier curves show percentage granulomas sterilized over 180 days of treatment with MXF, GFX or LVX (N = 210 granulomas).

Time to sterilization and percentage of granulomas sterilized are captured by Kaplan-Meier curves (Fig 7). Our simulations predict that MXF and LVX sterilize significantly higher percentages of granulomas compared to GFX.

Based on these data we conclude that the three FQs are similar, with MXF having a slight advantage over LVX (faster intracellular killing), and LVX a slight advantage over GFX (more granulomas sterilized).

### Uncertainty analysis supports predicted advantages of MXF over LVX and GFX

To determine the effect of parameter uncertainty on our efficacy predictions, we quantify the effect of individual parameters on model outputs, as described in Sensitivity Analysis in Methods and in [33]. Briefly, we simultaneously sample all antibiotic parameters in ranges spanning values for the three FQs (Table in S2 Table), and calculate partial rank correlation coefficients (PRCCs) between each parameter and model output (Table in S3 Table). Consistent with previous results [20], PRCCs reveal that plasma PK parameters (plasma clearance rate constant, *CL)*, tissue PK parameters (cellular accumulation ratio, *a*, and permeability coefficient, *PC*) and PD parameters (maximum intracellular activity, *E*_*max*,*BI*_, and C_50_ for intracellular Mtb, *C*_*50*,*BI*_) are drivers of infection and inflammation in the model. Outputs driven by these parameters include: bacterial load, macrophage activation, T cell activation, and TNF-α and IL-10 production. There is relatively low uncertainty in the plasma and tissue PK parameters, since they are estimated from calibration to multiple *in vitro* and *in vivo* data sets.

PRCCs indicate that uncertainty in intracellular PD parameters influences model outputs, while extracellular and non-replicating PD parameters do not (Table in S2 Table). This result is expected based on the concentration profiles in Fig 6. Intracellular bacteria are exposed to antibiotic concentrations close to the measured ranges for *C*_*50*,*BI*_, and therefore uncertainty in these values would affect predicted efficacy. I.e. if the *in vivo C*_*50*,*BI*_ values are significantly higher or lower than the *in vitro* measured values, the intracellular bacterial population would have a more or less significant role, respectively, in the long-term bacterial response to treatment in our simulations.

It is not currently possible to directly measure intracellular PD parameters *in vivo*, and we therefore rely on *in vitro* measurements. The importance of these parameters in our efficacy predictions highlight the need for controlled *in vivo* efficacy studies that would allow for indirect estimation of PD parameters through calibration of bacterial loads to per-granuloma experimental data. Nonetheless, unless one FQ’s PD parameters are more sensitive to *in vitro* conditions than the others, our conclusion of MXF having an advantage over LVX and GFX should still hold.

### Bacterial subpopulation-specific simulated EBA reflects clinical sputum EBA

EBA, defined as the daily decrease in sputum bacterial burden measured in colony forming units (CFU), is frequently used to assess the efficacy of a single drug in the first 7 or 14 days of treatment in clinical trials [34]. Here, we introduce a new term: ‘*in silico* EBA’, which is defined as the daily decrease in *simulated* bacterial burden per granuloma. *In silico* EBA is also calculated for individual bacterial subpopulations, e.g. the ‘*in silico* intracellular EBA’ is the daily decrease in simulated intracellular bacteria per granuloma. Compared to clinical EBA measured from sputum [8], *in silico* EBA is lower for all three FQs (Table 4). This is expected since our model tracks all bacteria in granulomas, whereas sputum samples contain a subpopulation of bacteria that may not fully represent the population in granulomas. This discrepancy between Mtb found in sputum vs. granulomas has been implicated in the poor ability of clinical sputum EBA to predict sterilization and long-term treatment outcomes [35]. *In silico* intracellular and extracellular replicating EBA more closely resemble clinical sputum EBA, compared to *in silico* non-replicating EBA, suggesting that intracellular and extracellular replicating subpopulations could be enriched in sputum. This is in agreement with human data showing high proportions of intracellular bacteria in sputum [36]. *In silico* intracellular EBA confirms that MXF is more efficacious than GFX and LVX. All three FQs have similar EBA against extracellular and non-replicating bacteria.

**Table 4:** EBA and extended EBA in clinical data and in simulations.

		Clinical (log_10_CFU/ml/day) [8]	Simulation (log_10_CFU/granuloma/day)			
			Total Mtb	Intracellular	Extracellular	Non-replicating
EBA (Day 0–2)	MXF	0.33 ± 0.39	0.19 ± 0.09	0.34 ± 0.15	0.30 ± 0.19	0.02 ± 0.06
	GFX	0.35 ± 0.27	0.11 ± 0.08	0.12 ± 0.10	0.26 ± 0.19	0.007 ± 0.06
	LVX	0.45 ± 0.35	0.14 ± 0.07	0.17 ± 0.11	0.33 ± 0.21	0.01 ± 0.06
Extended EBA (Day 2–7)	MXF	0.17 ± 0.09	0.06 ± 0.05	0.26 ± 0.14	0.15 ± 0.11	0.01 ± 0.01
	GFX	0.17 ± 0.13	0.06 ± 0.06	0.07 ± 0.05	0.18 ± 0.11	0.008 ± 0.02
	LVX	0.18 ± 0.13	0.07 ± 0.06	0.12 ± 0.08	0.16 ± 0.13	0.01 ± 0.02

Values show means ± standard deviation for 10 patients (clinical) and 210 granulomas (simulation).

### MXF treatment is more efficacious during patient non-compliance and treatment interruption

With any TB treatment there is a risk of patient non-compliance (inconsistent dosing throughout the treatment period) or treatment interruption (incomplete treatment), often arising from side effects and long treatment duration [37]. Our results show that both bacterial load and host immune responses decline more quickly during MXF treatment, compared to LVX and GFX treatment (Fig 5). This raises the question: is MXF treatment more sensitive to non-compliance or treatment interruption due to lower immune responses? Or, is the bacterial population sufficiently controlled by the antibiotic such that the lower inflammatory response does not affect infection control, even during non-compliance or treatment interruption?

**Fig 8:**
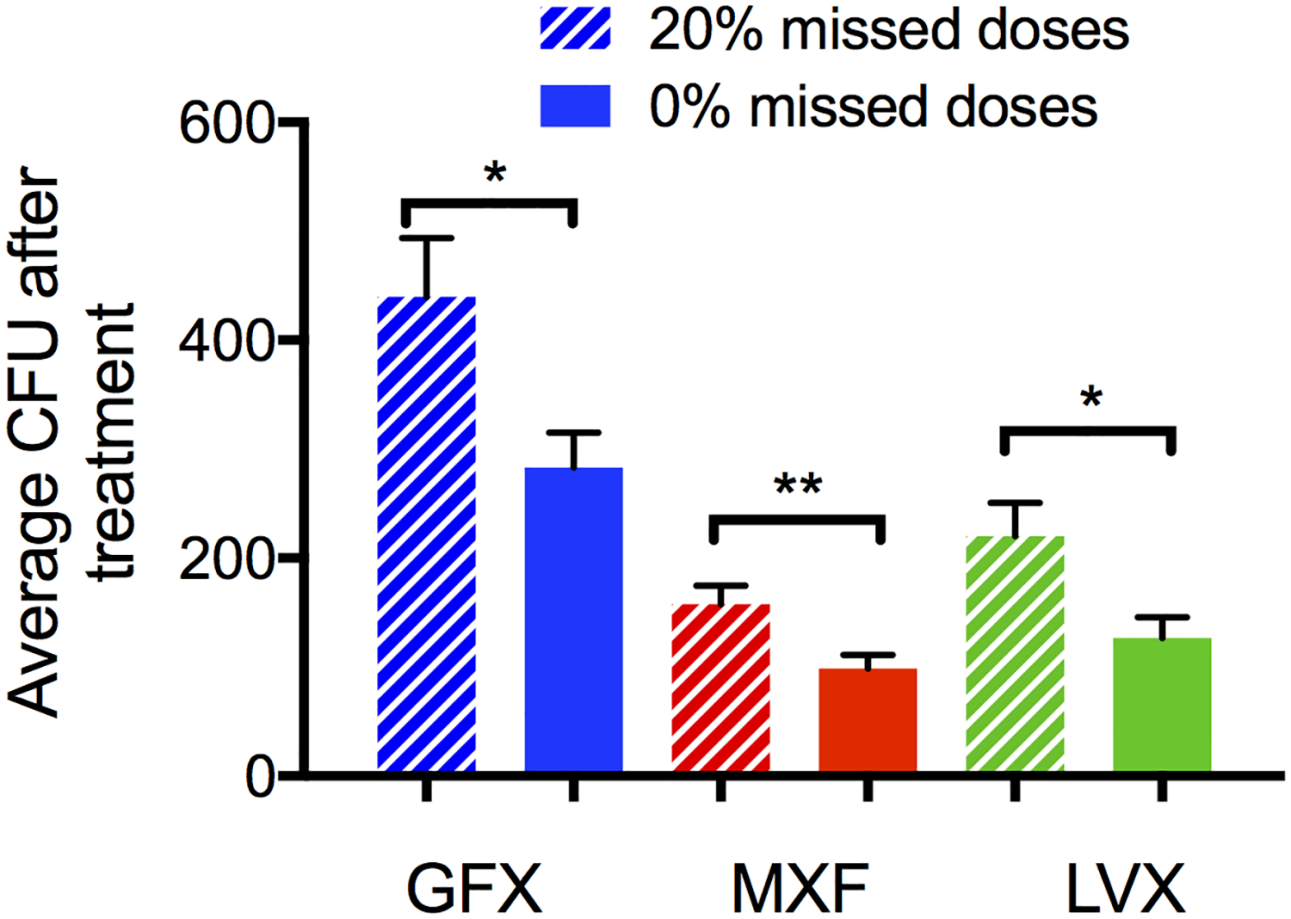
Simulated effects of non-compliance on bacterial load following treatment. Treatment simulations are repeated but programmed to randomly miss 20% of doses. Graph shows mean and standard error of total bacteria (CFU) after 180 days of treatment with MXF, GFX or LVX for 100% compliance (solid bars) or 20% missed doses (hashed bars). *: p-value < 0.05, **: p-value < 0.005.

**Fig 9:**
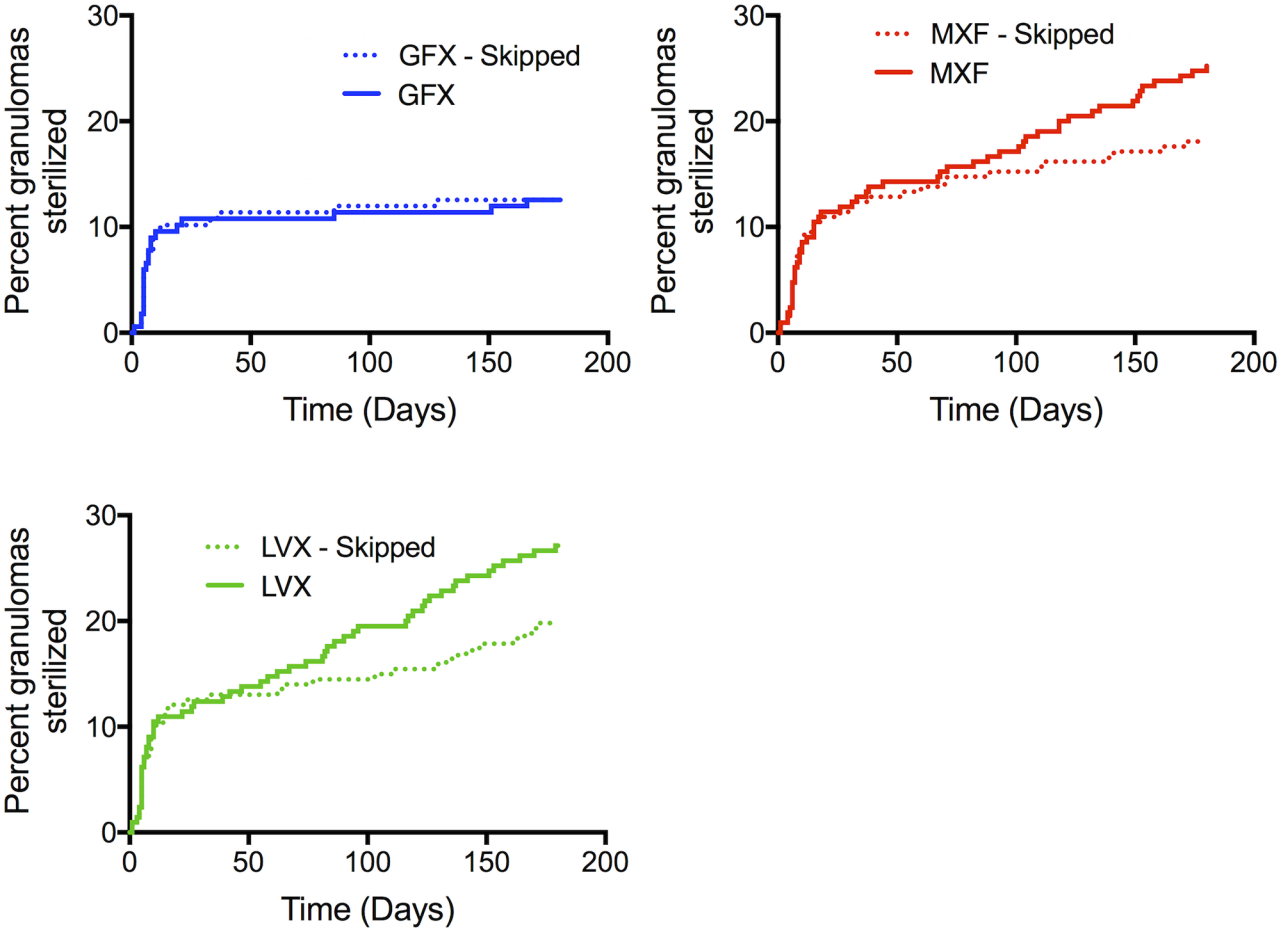
Simulated effects of non-compliance on granuloma sterilization. Percentage granulomas sterilized over 180 days of treatment with MXF, GFX or LVX (N = 210 granulomas), comparing 100% compliance (solid lines) to 20% missed doses (dotted lines) for each FQ.

We predict the efficacy of each FQ during non-compliance by simulating 6 months of daily therapy with random skipping of 20% of the doses. This threshold is chosen because it is commonly used to define patients as ‘compliant’ in clinical trials [38]. Bacterial load after treatment shows increases under non-compliance conditions for GFX, MXF and LVX therapy, relative to full compliance (Fig 8). However, bacterial loads during non-compliant MXF and LVX treatment is lower than fully compliant GFX treatment. Kaplan-Meier curves comparing each FQ in the compliant vs. non-compliant scenario show noticeable, but statistically insignificant, differences for LVX and MXF (Fig 9). However, percentage of granulomas sterilized during non-compliant conditions for MXF (18%) and LVX (20%) treatments is still higher than during fully compliant GFX treatment (13%). These small differences at the granuloma level could manifest as clinically significant if we consider that each patient likely has multiple granulomas [2, 39, 40]. For example, if a single granuloma has a 5% probability of failing treatment, a person with 10 granulomas has a 40% probability of failing treatment. Studies in non-human primates indicate they have on average 46 granulomas [41], so this is a significant factor.

**Fig 10:**
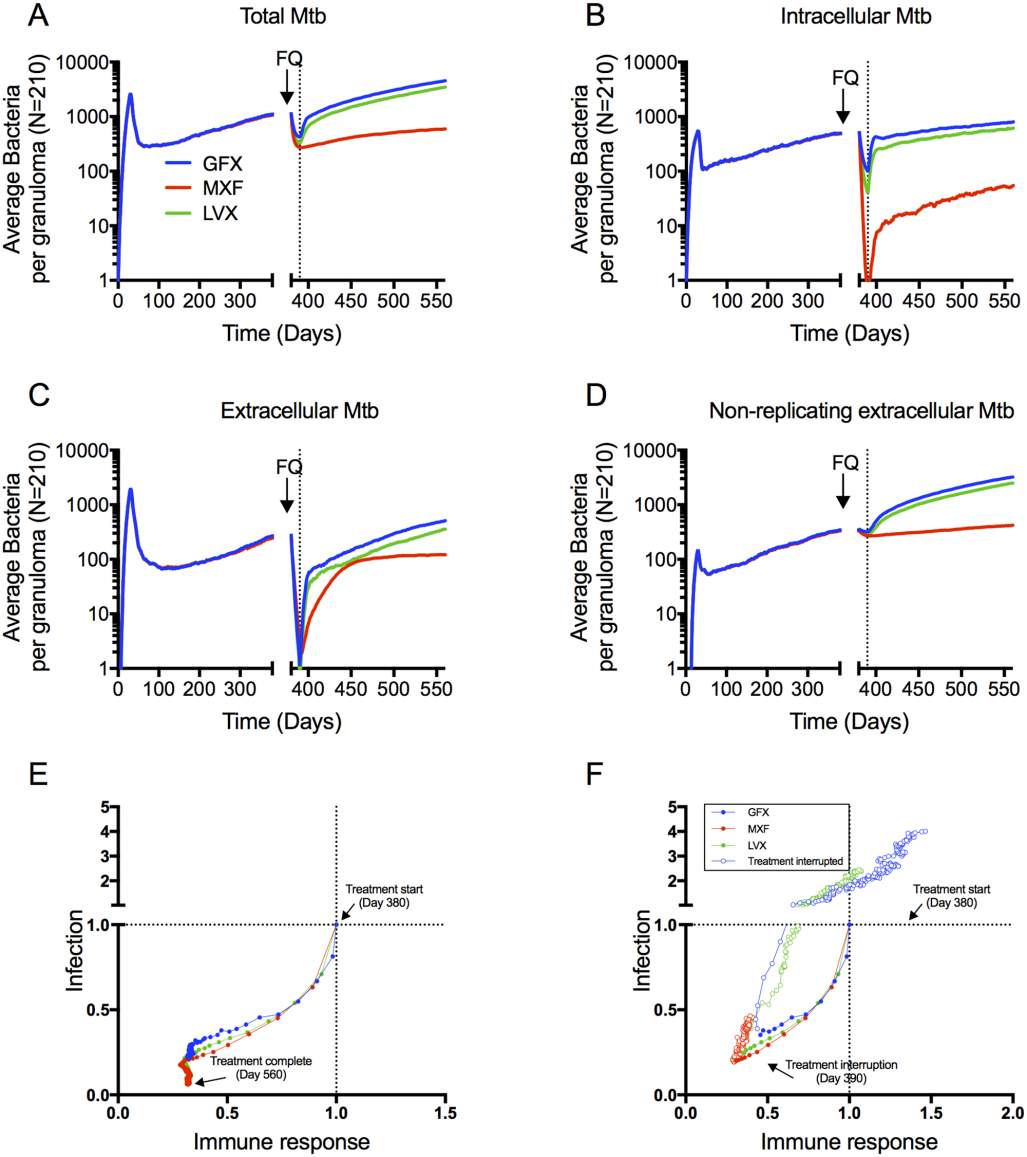
Bacteria in all subpopulations increase more slowly following MXF treatment interruption, compared to GFX and LVX (A-D). Lines show means of 210 *in silico* granulomas, with infection starting at day 0, daily FQ treatment starting at day 380 (arrows). Treatment is interrupted after 10 days (vertical dotted lines), and the simulation is continued to day 560 without antibiotics. (E-F) Immune score (x-axes) and infection score (y-axes) decrease during complete treatment (E) and rebound following treatment interruption after 10 days (F). The start of treatment is located at the intersection of the dotted lines. Filled circles indicate the treatment phase, and open circles indicated progression following treatment interruption.

We predict the efficacy of each FQ under treatment interruption by simulating 10 or 70 days of daily treatment, after which we stop treatment for the rest of the 6-month period. We choose 10 days based on clinical studies suggesting that treatment interruptions start increasing around 2 weeks of treatment [42], and our earlier results suggest that differences in immune response and bacterial load is most pronounced at this time. We choose 70 days for late interruption simulation time because at that point the immunological differences between FQs have largely disappeared (Fig 5E and 5F). Bacterial load shows sharp increases following interruption of GFX and LVX treatment, and a return to the pre-treatment trajectory for all bacterial subpopulations (Fig 10A–10D). In contrast, while interruption of MXF treatment results in an increase in all bacterial subpopulations, the increase is slower and the infection follows a slowed trajectory compared to pre-treatment. We can visualize the opposing forces of infection and immune responses by defining an immune score (a collective metric of immune response) and an infection score (a collective metric of infection severity). The immune score is defined as: (Activated macrophages* + Activated IFN-γ-producing T cells* + Activated cytotoxic T cells* + concentration of free TNF-α *)/4; where ‘*’ indicates that the value is normalized to its value at the start of treatment. Similarly, the infection score is defined as: (Infected macrophages * + Chronically Infected macrophages * + Extracellular Replicating Mtb* + Extracellular Nonreplicating Mtb*)/4. Tracking the immune and infection scores throughout treatment (Fig 10E and 10F) indicates that although the immune response is weaker during MXF treatment, it is sufficient to control the significantly reduced bacterial population. If treatment is interrupted after 70 days, the immune score and infection score remain stable following MXF and LVX interruption, while GFX interruption results in a slight increase in infection score (Table in S4 Fig).

Based on our *in silico* predictions that MXF-treated granulomas have lower bacterial loads and lower levels of treatment failure during non-compliance and treatment interruption, MXF is recommended over LVX and GFX in patients deemed at high risk of non-compliance or treatment interruption.

## Discussion

There are a number of new anti-TB antibiotics and antibiotic regimens in development and in various stages of clinical testing [43, 44]. To maximize the useful lifespan of new and existing antibiotics, we need to optimize their implementation. Efficacy of any antibiotic depends on a combination of factors (Table 1), and in a nonlinear and often non-intuitive way. The complexity stems from granuloma pathology, host dynamics, pathogen interactions and drug properties [4]. Systems pharmacology approaches that combine host, pathogen and antibiotic dynamics are ideal tools to study these complexities in a single model system, and are valuable in identifying promising treatment regimens to advance to animal and clinical studies.

We use a systems pharmacology approach to compare efficacy of three FQs in TB granulomas, concluding that MXF has a small but potentially clinically significant advantage over LVX, and LVX over GFX. MXF outperforms LVX and GFX in terms of total bacterial load, EBA and efficacy during non-compliance and treatment interruption. MXF and LVX each outperform GFX in terms of time to granuloma sterilization as well as percentage of granulomas sterilized. We would therefore recommend MXF over LVX, and LVX over GFX. Our predictions are currently being tested in rabbit models of TB, and will inform future studies in non-human primates. These studies will also be used to refine estimates of important *in vivo* intracellular PD parameters for future simulations. Our results could help guide FQ selection for MDR treatment as well as for future clinical trials for drug sensitive TB treatment.

In addition to recommendations for future treatment and trials, our work also provides insight into clinical trial results. Recent phase III clinical trials explored the possibility of using MXF or GFX to shorten the 6-month treatment regimens prescribed for drug-sensitive TB [45–47]. All three trials failed to show non-inferiority compared to the standard 6-month regimen. In contrast, preclinical results in mouse models of TB showed FQs can improve cure rates [48, 49] or bactericidal activity over shorter time scales [50, 51]. Phase II clinical studies showed a larger decline in sputum bacterial load when FQs are substituted into the standard regimen [18, 52, 53], but the 8 week time points evaluated in these phase II studies did not predict long term outcomes such as sterilization and recurrence that appeared in the phase III studies [45]. Our computational approach could explain why MXF and GFX failed to improve treatment outcomes in these 4-month regimens. In previous studies we found that granulomas that fail to sterilize with INH and RIF treatment contain mostly intracellular and non-replicating Mtb [20]. Our results here indicate that GFX and MXF would be unable to sterilize the non-replicating bacterial subpopulation. Taken together, these results suggest that MXF and GFX would be complementary to INH or RIF in the tested 4-month regimens by targeting the intracellular populations not eliminated by INH or RIF. However, the non-replicating populations that survive INH or RIF in the 4-month regimens would still persist in the context of MXF or GFX. Our results therefore predict that non-inferiority of these 4-month regimens could be due to non-replicating bacteria that persist throughout therapy, contributing to subsequent relapse.

Toward the goal of optimizing TB treatment regimens that rely on multiple drugs, in future simulations we will explore the performance of these FQs in combination with INH and/or RIF and other anti-TB antibiotics. Combination therapy presents a number of challenges that can be studied using computational approaches. The ability of our method to track responses of different bacterial subpopulations to treatment will allow us to design and optimize combination therapies that effectively target all bacterial subpopulations. One particular challenge we can address is predicting the risk of ‘effective monotherapy’. Effective monotherapy occurs when spatial or temporal windows of monotherapy arise, even under combination therapy, due to PK differences between the antibiotics given and could lead to inadvertent selection of drug resistant bacteria [19, 54, 55]. Beyond windows of effective monotherapy, optimizing multi-drug therapy is complicated by drug-drug interactions. The inherent properties of antibiotics (e.g. how they are metabolized) can result in complex networks of interaction (synergy/antagonism)[56, 57] that also influence the selection of drug resistant bacteria [58]. While such detailed interaction networks are not currently available for Mtb, work in *Mycobacterium marinum* [57] could inform future optimization studies.

We predict that FQ concentrations inside granulomas must be at least 3-fold higher than those simulated here to eliminate the non-replicating bacterial population. Indeed simulations with higher doses predict granuloma sterilization within 20 days (data not shown). These doses would likely result in toxicity [59]. Targeted or inhaled drug delivery strategies could be used to increase the concentration within granulomas while lowering systemic distribution and therefore toxicity [22, 60]. However, PK of antibiotics after release from such targeted delivery vehicles determines the feasibility of such approaches. For example, we previously predicted that INH is suitable for inhaled delivery, but RIF’s PK would require unrealistically high carrier loadings [22]. It is difficult to anticipate whether inhaled delivery would be a feasible alternative to oral dosing for FQs based on their plasma and tissue PK parameters derived here. Future studies would have to systematically explore the delivery vehicle parameter space to answer this question.

EBA is a treatment outcome metric commonly used to compare antibiotic efficacy in early clinical trials, but the EBA has a poor ability to predict sterilization and long-term treatment outcomes [35, 61]. The inability of bacterial load measurements in sputum to capture bacterial dynamics inside granulomas is supported by our model results that show lower *in silico* EBA compared to clinical sputum EBA. Systems pharmacology approaches could help extend the impact of EBA studies by predicting underlying bacterial dynamics.

As with all computational models, the necessary assumptions made in our model place limitations on the predictions presented here. Due to the complexity of the system, the model has a large number of parameters that are known with varying degrees of certainty. When possible, we increase our confidence in parameter values by fitting to multiple experimental datasets (e.g. LCMS and MALDI-MSI to estimate diffusivity) and by including inter-individual variation in the calibration and prediction simulations. *In vivo* efficacy studies can assess the effect of these limitations on our current predictions, and help lessen these effects for future predictions.

Systems pharmacology provides a platform to integrate sometimes-conflicting experimental data with computational modeling to further our understanding of PK/PD interactions in an *in vivo* setting. It also allows us to narrow the design space of combinations of drugs to better determine optimal treatments for patients. Here we have focused on FQs, but the approach can be applied in any setting of drug distributions in specific tissues. In TB, multiple drug regimens are used for long periods of time and thus the next necessary step to increasing our understanding is to perform virtual clinical trials that will allow us to predict the right combinations of drugs to shorten treatments.

## Materials and methods

### Ethics statement

All animal studies were carried out in accordance with the Guide for the Care and Use of Laboratory Animals of the National Institutes of Health, with approval from the Institutional Animal Care and Use Committee of the New Jersey Medical School, Newark, NJ, and the National Institute of Allergy and Infectious Diseases (National Institutes of Health), Bethesda, MD.

### In vitro and in vivo experimental data

The experimental data we obtained for model calibration is outlined here, and detailed below. *In vitro* pharmacodynamic data comprise dose response curves in liquid culture media [27] as well as in Mtb-infected bone marrow derived macrophages from C57Bl/6 mice. *In vitro* pharmacokinetic data comprise drug uptake in human THP-1 cells and caseum binding.

*In vivo* pharmacokinetic studies in rabbits include: temporal LC/MS-MS of antibiotic concentrations in plasma and homogenized granulomas between 0.5 and 24 hrs following dosing of each FQ; and MALDI-MSI (matrix-assisted laser desorption ionization—mass spectrometry imaging) providing semi-quantitative images of antibiotic spatial distribution within granulomas.

### Experimental methods

#### Dose response curves

MIC in liquid culture was determined as described in [27]. To measure intracellular MIC, bone marrow derived macrophages from C57Bl/6 mice were treated with interferon gamma overnight. Macrophages were infected with Erdman-Mtb containing a luciferase expressing plasmid at a multiplicity of infection of 10. After 4 hours of infection extracellular bacteria was washed off. Media was supplemented with drug. Luminescence readings were taken Day 0, Day 1, Day 2, and Day 3.

#### Drug uptake assay in human THP-1 cells

Human macrophage-like THP-1 cells (ATCC TIB-202) were maintained in RPMI 1640 medium (1× without L-glutamine, Corning Cellgro 15-040-CV) supplemented with 10% FBS and 2 mM L-glutamine (Sigma G7513) at 37°C in a 5% CO2 incubator. THP-1 cells were initiated at a density of 2 × 10^5^ to 4 × 10^5^ cells/ml in 75 cm^2^ flasks. After 3 days of incubation, the number of viable cells was counted using the trypan blue exclusion protocol (http://www.lifetechnologies.com/us/en/home/references/gibco-cell-culture-basics/cell-culture-protocols/trypan-blue-exclusion.html) and diluted to 1 × 10^6^ cells/ml. Phorbol 12-myristate 13-acetate (PMA) was added to a final concentration of 100 nM, and 1 × 10^5^ cells were seeded into each well of 96-well tissue culture-treated plates (Greiner Bio One, 50-823-592). Plates were incubated overnight at 37°C in a 5% CO_2_ incubator to allow cells to attach to the bottom of the wells. After overnight incubation, cells were gently washed twice with equal volume of PBS to remove unattached and dead cells.

Old medium was removed carefully, and medium supplemented with MXF, GFX or LVX at final concentrations of 4, 2 and 16μM respectively, was added. Cells were incubated at 37°C under normal atmospheric conditions. After 30 min incubation, media were removed carefully, and cells were gently washed twice with equal volume of ice-cold PBS to remove any extracellular drug residuals. Cells were then lysed with equal volume of deionized water for 1 h at 37°C. Lysates were transferred to 1.5-ml centrifuge tubes and analyzed right away. The number of cells per well was estimated by staining with the DNA-binding fluorescent dye PicoGreen, using half of each sample while the rest of the lysate was analyzed by liquid chromatography coupled to tandem mass spectroscopy as described previously [62]. An average macrophage diameter of 11.3 μm was measured by confocal microscopy and used to infer intracellular concentrations. The absolute intracellular/extracellular ratio is influenced by the macrophage diameter used in the ratio calculation. However, the computational model accounts for this influence since we use results from the uptake assay to identify feasible *ranges*, and then calibrate granuloma PK to an independent LC/MS-MS dataset measuring concentrations in granulomas. Experimental results are summarized in Table 5.

**Table 5:** *In vitro* fluoroquinolone properties.

	Uptake into human cells		
Fluoroquinolone	Intracellular-to-extracellular ratio at 0.5 h	Incubation concentration (μM)	Caseum unbound fraction (%) (mean, SD)
MXF	4.35±0.25	4	13.5, 3.67
GFX	2.78±0.44	2	16.3, 4.2
LVX	2.09±0.29	16	17.66, 2.03

Uptake into human cells, measured as intracellular-to-extracellular concentration ratio. Caseum unbound fraction as measured by RED assay.

#### Caseum binding

Specific pathogen–free, individually housed female New Zealand White (NZW) rabbits were used for aerosol infection by *Mtb* HN878 or *M*. *bovis* AF2122, as previously described [63, 64]. Briefly, rabbits were exposed to *Mtb*–or *M*. *bovis*–containing aerosol using a nose-only delivery system. Three hours after infection, three rabbits were euthanized, and serial dilutions of the lung homogenates were cultured on Middlebrook 7H11 agar plates to enumerate the number of bacterial colony forming units (CFUs) implanted in the lungs. The infection was allowed to progress for 4 weeks (*M*. *bovis*) or 12–16 weeks (*Mtb*) before necropsy and collection of cavity caseum.

The rapid equilibrium dialysis (RED) device comprises two side-by-side chambers separated by a vertical cylinder of dialysis membrane (molecular weight cut-off ~8,000)[65] Caseum samples were diluted tenfold in PBS and homogenized. Drug stock solutions were added to the homogenized samples to give the final concentration of 5 μM (<1% DMSO). The inserts were placed in the open wells of the Teflon base plate. 200 μl spiked caseum was placed in the sample chambers and the buffer chambers were filled with 350 μl PBS. The plates were sealed and incubated at 37°C for 4 h on an orbital shaker set at 200 rpm. Following incubation, 50 μl caseum from the sample chamber was transferred to tube containing 50 μl PBS. Similarly, 50 μl PBS was transferred from the buffer chamber to a tube containing 50 μl neat caseum. 400 μl organic solvent mixture (30/70 methanol/ACN with internal standard) was added to each sample. Samples were vigorously vortexed and centrifuged. The supernatant was transferred to 96-well plates and stored at −20°C.

Fraction unbound (*f*_*u*_) in undiluted caseum was calculated using Eq 1, where the dilution factor (D) is equal to 10. Recovery (mass balance) for each assay was calculated using Eq 2.

$$\text{Undiluted }f_{u}=\frac{1/D}{\left(\left(\frac{1}{fu}\right)\;-\;1\right)\;+\;1/D}$$ (1)

$$\text{Recovery }=\frac{mass\;in\;sample\;chamber+mass\;in\;buffer\;chamber}{mass\;in\;sample\;chamber\;at\;t=0}\times 100\%$$ (2)

Results are summarized in Table 5.

#### Plasma and granuloma pharmacokinetic studies in rabbits—LC/MS-MS

In pharmacokinetic studies, specific pathogen-free, individually housed female NZW rabbits, weighing 2.2 to 2.6 kg, were used for aerosol infection by *Mtb* HN878, as previously described [63]. HN878 was selected since it is known to generate a representative range of human-like granulomas in infected rabbits. Briefly, rabbits were exposed to *Mtb*-containing aerosol using a nose-only delivery system. Three hours post-infection, three rabbits were euthanized, and serial dilutions of the lung homogenates were cultured on Middlebrook 7H11 agar plates to enumerate the number of bacterial colony forming units (CFUs) implanted in the lungs. The infection was allowed to progress for 12 to 16 weeks.

In plasma pharmacokinetic studies, groups of 3 to 8 infected rabbits received between 14 and 21 doses of each fluoroquinolone as indicated (Table 6). Blood samples were collected in EDTA-coated tubes pre-dose and at various time points up to 24h post-dosing. Samples were inverted 8 to 10 times and kept on ice for no more than 10 minutes prior to centrifugation at 2000-3000g units for 10 minutes to recover plasma and quantify each drug by LC/MS-MS.

**Table 6:** Summary of plasma and granuloma pharmacokinetic studies (dose size, number of samples, number of doses and time points) by FQ and experimental method.

	Plasma PK	Granuloma PK	
	LC/MS-MS	LC/MS-MS	MALDI-MSI
MXF	Dose size: 25 mg/kg Samples: N = 4 rabbits Doses: 21 doses Time points: 0, 0.5, 1, 2, 4, 7, 24 hours post dose Dose size: 60 mg/kg Samples: N = 8 rabbits Doses: 21 doses Time points: 0, 0.5, 2, 4, 7, 24 hours post dose	Dose size: 100 mg/kg Samples: N = 7 rabbits (up to 29 samples per rabbit) Doses: 1 dose Time points: 2, 6, 12, 19.5, 24 hours post dose	Dose size: 100 mg/kg Samples: N = 2 rabbits (2 or 3 samples per rabbit) Doses: 1 doses Time points: 2, 6 hours post dose
GFX	Dose size: 40 mg/kg Samples: N = 3 rabbits Doses: 15 doses Time points: 0, 0.5, 1, 2, 4, 6, 8, 24 hours post dose	Dose size: 100 mg/kg Samples: N = 9 rabbits (up to 33 samples per rabbit) Doses: 1 dose Time points: 2, 6, 24 hours post dose	Dose size: 100 mg/kg Samples: N = 6 rabbits (1 or 2 samples per rabbit) Doses: 1 doses Time points: 2, 6, 24 hours post dose
LVX	Dose size: 65 mg/kg Samples: N = 3 rabbits Doses: 14 doses Time points: 0, 0.5, 1, 2, 4, 6, 8, 24 hours post dose Dose size: 100 mg/kg Samples: N = 4 rabbits Doses: 14 doses Time points: 0, 0.5, 1, 2, 4, 6, 8, 24 hours post dose	Dose size: 75 mg/kg Samples: N = 16 rabbits (up to 36 samples per rabbit) Doses: 1 dose Time points: 2, 6, 12, 16, 24 hours post dose	Dose size: 75 mg/kg Samples: N = 3 rabbits (1 to 4 samples per rabbit) Doses: 1 doses Time points: 2, 6 hours post dose

In granuloma pharmacokinetic studies, groups of 7 to 16 infected rabbits received a single dose of fluoroquinolone as indicated (Table 6). Individual lung granulomas and pieces of uninvolved lung tissue were weighed and homogenized in approximately—but accurately recorded—5 volumes of phosphate buffered saline (PBS). Homogenization was achieved using a FastPrep-24 instrument (MP Biomedicals) and 1.4mm zirconium oxide beads (Precellys). Proteins were precipitated by adding 9 volumes of 1:1 acetonitrile:methanol containing 0.5μg/ml of deuterated moxifloxacin (Toronto Research Chemicals, Inc) as internal standard to 1 volume of plasma or homogenized tissue sample. The mixtures were vortexed for 5min and centrifuged at 4,000rpm for 5min. The supernatant was then transferred for LC/MS-MS analysis. LC/MS-MS analysis was performed with an Agilent 1260 system coupled to an AB Sciex 4000 Q-trap Mass Spectrometer (positive mode electrospray ionization), and an Agilent Zorbax SB-C8 column (2.1 x 30 mm, 3.5 μm particle size), with the column temperature fixed at 24°C. A 4 min reverse phase gradient was used with 0.1% formic acid in Milli-Q water (mobile phase A) and 0.1% formic acid in acetonitrile (mobile phase B). Injection volumes were routinely 1μL. The Mass Selective Detector was set to MRM (multiple reaction monitoring) mode using positive electrospray ionization, monitoring for the ions of interest (m/z 402.18/358.10 for MXF; m/z 362.02/318.50 for LVX; m/z 376.00/261.20 for GFX). The internal standards were Moxifloxacin–d4, Levofloxacin–d8 and verapamil (m/z 455.40/165.20). The lower limits of quantification were 1ng/mL for MXF and LVX and 5ng/mL for GFX.

#### Spatial granuloma pharmacokinetic studies—MALDI-MSI

MALDI-MSI (matrix-assisted laser desorption ionization—mass spectrometry imaging) analysis was performed using a MALDI LTQ Orbitrap XL mass spectrometer (Thermo Fisher Scientific, Bremen, Germany) with a resolution of 60,000 at m/z 400, full width at half maximum. The resolution was sufficient to resolve the drug and respective labeled standards peaks from background without the requirement for MS/MS and subsequent loss of signal. However, drug peak identities were confirmed by acquiring several MS/MS spectra directly from the dosed tissues.

Spectra were acquired in positive mode and with a mass window of m/z 200–600. This range covered the three fluoroquinolones and any potential salt adduct peaks. A laser energy of 10μJ was applied, and 5 laser shots were fired at each position (total of 1 microscan per position). The laser step size was set at 75 μm, at which small necrotic areas within granulomas could easily be resolved and no overlapping of the laser spot on adjacent acquisitions was observed.

Data visualization was performed using Thermo ImageQuest software. Normalized ion images of MXF were generated by dividing MXF [M+H]^+^ signal (*m/z* 402.182 ± 0.003) by MXF-d3 [M+H]^+^ signal (*m/z* 405.201 ± 0.003). Normalized ion images of GFX were generated by dividing GFX [M+H]^+^ signal (*m/z* 376.167 ± 0.003) by GFX-d3 [M+H]^+^ (m/z 380.192). Normalized ion images of LVX were generated by dividing LVX [M+H]^+^ signal (*m/z* 362.151 ± 0.003) by LVX-d3 [M+H]^+^ signal (*m/z* 365.170 ± 0.003).

Relative quantitation of MXF, LVX and GFX within caseum and cellular granuloma areas was performed using ImaBiotech Software Quantinetix^™^(v 1.7, Loos, France). ROIs were drawn by first aligning and superimposing the MS image over the optical scan of the tissue. The MS image layer was made transparent and the ROIs were drawn based upon the optical scan and by referral to an adjacent H&E-stained tissue section as a guide to avoid bias in region selection.

### Computational model implementation

#### In silico lung granulomas

To simulate granuloma formation and function, we use our computational model, *GranSim* [23–26] (Fig 1). *GranSim* is a hybrid model integrating an agent-based model (ABM) with ordinary (ODE) and partial (PDE) differential equations. Pseudo-code detailing the rules, equations and implementation of *GranSim* can be found at malthus.micro.med.umich.edu/GranSim/. A model executable, along with instructions and parameter files to run FQ treatment, are available at malthus.micro.med.umich.edu/lab/supplements/Fluoroquinolone/.

*GranSim* is an established model that has been calibrated to extensive data from a non-human primate model of TB [20, 22–26]. *GranSim* is implemented as described in these references and pseudo-code, with the following updates made in this work:

1. Inclusion of fluoroquinolone dynamics (previous versions include isoniazid and rifampin).
2. Dynamic representation of cellular uptake of antibiotics (previous versions assume pseudo-steady state)
3. Antibiotic binding to caseum and normal lung tissue (previous versions approximate binding by effective diffusivity parameters)

Updates 2 and 3 were necessary to reproduce experimental tissue PK data (LCMS and MALDI-MSI), and are detailed below.

Model outputs are both temporal and spatial. *GranSim* tracks events at the molecular scale in seconds (e.g. diffusion, binding, etc); at the cellular scale in minutes (e.g. phagocytosis, apoptosis); and at the tissue scale in days and months (e.g. granuloma formation, antibiotic activity). *GranSim* tracks host immune cells on a two-dimensional simulation grid of micro-compartments, representing a 2mm x 2mm section of lung tissue, and implements cellular movement and interactions according to a set of biology-based rules. Some micro-compartments are designated ‘vascular source micro-compartments’ (VSMs) where antibiotics and host immune cells enter the simulation grid from the blood. *GranSim* simulations are initiated by randomly placing VSMs and resident macrophages throughout the grid and adding a single infected macrophage at the center of the grid. Immune mechanisms in *GranSim* include: host cell recruitment (macrophages, T-cells), secretion and diffusion of chemokines and cytokines, cell activation and movement, phagocytosis of Mtb, host cell bursting due to Mtb growth, killing of Mtb by macrophages, and caseation in response to host cell death. ODEs describe receptor-ligand binding, internalization and signaling for cytokines (TNF-α, IL-10. PDEs describe diffusion on the simulation grid. *GranSim* is continually curated and extensively calibrated to reflect bacterial load, granuloma size and structure observed in non-human primates [20, 66, 67]. Emergent behavior of the system is the formation of *in silico* lung granulomas (Fig 1B).

#### In silico antibiotic distribution and activity

*GranSim* includes pharmacokinetic (PK) and pharmacodynamic (PD) mechanisms of antibiotics (Fig 1A). *GranSim* has been used previously to predict efficacy of isoniazid (INH) and rifampin (RIF) treatment [20–22]. Here for the first time we add the capability to treat with three fluoroquinolones (MXF, LVX, GFX). The PKPD model includes a plasma PK model, vascular permeation of antibiotics from plasma into lung tissue, diffusion in lung tissue, penetration into granulomas, accumulation inside host cells, and antibacterial activity. Our PK and PD models are detailed below. Model parameters are defined in Table 3 by action and FQ.

#### Plasma PK model

Plasma PK are modeled by a system of ODEs representing a two-compartment model with first-order absorption. Inter-individual variability is captured in the model by sampling plasma PK parameters (absorption rate constant, *k*_*a*_; intercompartmental clearance rate constant, *Q*; plasma volume of distribution, *V*_*p*_; peripheral volume of distribution, *V*_*pe*_; plasma clearance rate constant, *CL*) from a lognormal distribution. Antibiotic dynamics in the plasma compartment (*C*_*p*_, mg/kg) and peripheral compartment (*C*_*pe*_, mg/kg) are captured by:

$$\frac{dC_{p}}{dt}=k_{a}Dose-Q\left(\frac{C_{p}}{V_{p}}-\frac{C_{pe}}{V_{pe}}\right)-CL\frac{C_{p}}{V_{p}}$$

$$\frac{dC_{pe}}{dt}=\;Q\left(\frac{C_{p}}{V_{p}}-\frac{C_{pe}}{V_{pe}}\right)$$

#### Tissue PK model

Tissue PK is modeled as in [20–22] to capture antibiotic permeation through vascular walls and diffusion within lung tissue. Based on new experimental data, we add in this work: dynamic representation of cellular uptake of antibiotics (previous versions assume pseudo-steady state); and antibiotic binding to caseum and normal lung tissue (previous versions approximate binding by effective diffusivity parameters). A set of ODEs describes the dynamics of antibiotics within each simulation grid microcompartment based on the local environment (e.g. amount caseation, presence of macrophage). The concentrations of free (*C*_*f*_), caseum-bound (*C*_*c*_), epithelium bound (*C*_*e*_) and intracellular (*C*_*i*_) antibiotics are described by:

$$\frac{dC_{f}}{dt}=k_{rc}C_{c}+k_{re}C_{e}+k_{out}C_{i}-\left(k_{fc}c+k_{fe}+k_{in}\right)\;C_{f}$$

$$\frac{dC_{c}}{dt}=k_{fc}cC_{f}-k_{rc}C_{c}$$

$$\frac{dC_{e}}{dt}=k_{fe}C_{f}-k_{re}C_{e}$$

$$\frac{dC_{i}}{dt}=k_{in}C_{f}-k_{out}C_{i}$$

Parameters are defined in Table 3, and rate constants are calculated from equilibrium constants: $k_{rc}=k_{fc}\left(\frac{f_{u}}{1-f_{u}}\right)$; kre=kfeKa; kin=akout. Caseation (*c)* represents the level of dead cellular debris (in arbitrary caseum units, *cu*). Caseation occurs in the model when the number of host cell deaths in a given microcompartment reaches a threshold. The number of cell death events in each grid microcompartment is used to approximate the level of caseation available for antibiotic binding.

#### Pharmacodynamics model

PD are implemented as in previous work using an Emax model [20–22]. Briefly, the killing rate constant is calculated as follows:

$$k_{kill,x}=E_{max}^{x}\frac{C_{x}^{H}}{C_{x}^{H}+C_{50,x}^{H}}$$ (3)

where x denotes the state of bacteria (intracellular, extracellular replicating or extracellular non-replicating), and parameters are defined in Table 3.

#### Model implementation

The simulation environment (*GranSim)* consists of portable C++ code currently targeted at Linux, Mac OS/X and Windows. There is a single threaded command line version used for parameter sweeps and a multi-threaded GUI version used for generating graphics of a single simulation. We use the Boost library for general C++ functionality, openGL and ffmpeg libraries for graphics and video and the Qt application framework for GUI, threading and open GL support.

Due to the stochastic nature of the model and differences in computational systems, simulation runtimes vary. Generation of control granulomas used to evaluate treatment has runtimes of ~3 hours per granuloma to simulate 200 days of infection. Treatment simulations have runtimes of ~9 hours per granuloma for 180 days of treatment.

### Model calibration

Parameters used in the model are known with varying degrees of certainty depending on the process that the parameters describe and if this process is experimentally measurable. Estimation of specific parameters is discussed in more detail below, but briefly we take a combination of the following approaches:

1. Parameter sweeps—we systematically and simultaneously sample the multi-dimensional parameter space to determine the influence of parameter uncertainty on model outcomes.
2. Different experimental metrics for one property—e.g. to estimate diffusivity, permeability etc. we calibrate the model to both LCMS and MALDI-MSI measurements of antibiotic concentrations within granulomas.
3. *In vitro* or literature measurements guide parameter sweeps—e.g. to estimate caseum unbound fraction we use *in vitro* data to define the parameter range used in tissue PK calibration; or to estimate the diffusivity we use literature estimates of diffusivity of small molecules in tumors to define the parameter range used in tissue PK calibration.

A summary of experimental data and how they are integrated into our computational framework is given in Table 2.

#### Plasma PK calibration

Plasma PK parameters are fit in SimBiology (MATLAB 9.1). We use a non-linear mixed effects model with a stochastic Expectation-Maximization (EM) algorithm assuming a constant error model and no covariates. The algorithm is run for 5 burn-in iterations, followed by 400 iterations, and all parameter estimates converged prior to reaching the iteration limit. Fitting is repeated three times for each FQ and the parameter set with the smallest Akaike Information Criterion (AIC) is selected. Rabbit parameters are fit to plasma concentrations measured as described above). Human parameters are fit to data from [9], extracted using WebPlotDigitizer [68] for MXF and GFX (400 mg) and LVX (1000 mg), with N = 9 or 10. To compare plasma PK parameters between rabbit and human data, we used a two-compartment plasma PK model for both data sets resulting in different parameter values than those for the one-compartment model used in [9].

#### Tissue PK calibration—Generating in silico granulomas

To calibrate the model to FQ measurements from granuloma PK studies (Table 2), we establish a set of *in silico* tissue samples. We generate a collection of 100 parameter sets, capturing inter-individual variation by randomly sampling host immune parameters and plasma PK parameters using LHS [33, 69, 70]. LHS ensures evenly distributed, simultaneous sampling of a multi-dimensional parameter space [33, 69]. The host parameter ranges used in this work (Table in S1 Table) is based on previous calibration to non-human primate data [20, 21].

We generate three *in silico* tissue samples for each parameter set: cellular granuloma, caseous granuloma, and uninvolved lung (the latter in the form of an uninfected simulation grid). We therefore treat three *in silico* tissue types for every parameter set (3 tissue types x 100 parameter sets = 300 samples).

#### Tissue PK—Calibrating to granuloma pharmacokinetic studies (LC/MS-MS and MALDI-MSI)

We estimate tissue PK parameters by calibrating model predicted FQ concentrations to the datasets described in Table 6. We sample the tissue PK parameter space 1500 times using LHS. The range that we search for each parameter is determined by *in vitro* data (where available) or from literature estimates (Table in S1 Text). When no experimental or literature estimates are available, wide ranges are used for calibration. Each of the 1500 parameter sets is tested in the 300 *in silico* tissue samples described above. For *each* of the 1500 tissue PK parameter sets, we simulate 24 hours of FQ dynamics in the 300 *in silico* tissue samples described above, following a single dose corresponding to the experimental doses.

We select the final parameter sets for tissue PK out of these 1500 in two phases. First, ten candidate tissue PK parameter sets are selected for each FQ that have the lowest sum of squared errors between the average of the simulations and average of the experimental LCMS data in caseous and cellular granulomas. Second, each of the ten candidate parameter sets is compared to MALDI-MSI data. The final candidate parameter set is selected manually from the 10 candidates where selection criteria are based on qualitative results from MALDI-MSI data. MALDI-MSI-derived criteria are: for MXF, we select a parameter set that gives lower concentration in caseum than in surrounding cellular areas, but with relatively uniform distributions throughout cellular areas; for GFX, a parameter set that gives lower concentration in caseum, but with some accumulation in cellular areas immediately surrounding the caseum; and for LVX a parameter set that gives distribution in cellular and caseous areas of granulomas. Note that a direct comparison between our simulations and MALDI-MSI is not possible due to the semi-quantitative nature of the MALDI-MSI data. Final estimates of tissue PK parameters fit in this work are listed in Table 3.

#### PD—Parameter estimation

Antibiotic efficacy in the model depends on local antibiotic concentrations as well as on bacterial location (intracellular, extracellular or in caseum). We use nonlinear least squares analysis to estimate PD parameters (*E*_*max*_, *C*_*50*_, *H*) from dose response curves in liquid culture (representing extracellular killing), in oxygen or nutrient limited conditions (representing killing in caseum) as well as in C57Bl/6 mouse bone marrow-derived macrophages (representing intracellular killing) (Figure in S1 Fig). *E*_*max*_ is maximum antibacterial activity, *C*_*50*_ is the concentration where 50% of maximum activity is achieved, and *H* is the Hill constant describing steepness of the dose response curve.

The mathematical model used to estimate *in vitro* bacterial growth and killing by antibiotics is used as in [20, 21, 71]. The Mtb population (*B*) changes due to growth and antibiotic killing according to:

$$\frac{dB}{dt}=\left(g-k_{kill}\right)B$$ (4)

where *g* is the growth rate constant and *k*_*kill*_ is the antibiotic killing rate constant (Eq 3).

We solve Eq (4) assuming a growth rate constant (*g*) of 0.69/day, corresponding to a 24 hr generation time [72, 73]. We calibrate model-predicted bacterial levels (*B*) to experimental data available at 5 days for liquid culture experiments and 3 days for mouse macrophage experiments using nonlinear least squares analysis. Estimated parameters are given in Table 3, and fitted dose response curves are shown in Figure in S1 Fig.

### Sensitivity analysis

To quantify the influence of individual antibiotic parameters on model outcomes, we perform a sensitivity analysis (SA). We sample all antibiotic parameters simultaneously and uniformly using Latin hypercube sampling (LHS) [33, 69, 70]. Parameters and ranges used for SA are listed in Table in S2 Table. Parameters were sampled 400 times, and each set of parameters was simulated in six unique granulomas. Simulations consisted of daily treatment between 380 and 410 days post infection with a dose of 400 mg/kg. Model sensitivity is quantified using Partial Rank Correlation Coefficients (PRCC) between model parameters and model outputs. Model outputs are averaged over the six granulomas tested. A p-value < 0.01 is considered significant for PRCC.

### Treatment simulation

#### In silico antibiotic treatment

To compare FQ efficacy, we use *GranSim* to generate a new repository of 210 *in silico* granulomas. This repository contains a spectrum of granuloma types with varying sizes, caseation levels and bacterial loads, reflective of non-human primate granulomas [2, 39, 74, 75]. Granuloma variation is obtained as for tissue PK calibration granulomas (Table in S1 Table). We start treatment in these granulomas 380 days post infection, by initiating 6 months of daily FQ monotherapy with 400mg of MXF or GFX, or 1000mg of LVX according to clinical recommendations [76]. As an example of a non-compliance situation, we simulate random skipping of 20% of doses over 180 days of treatment. This pattern of non-compliance is consistent with electronic dose monitoring data [77]. As an example of treatment interruption we treat daily for 10 days, and then stop dosing for the rest of the 180 days.

#### Treatment outcome metrics

We use several metrics to compare treatment outcomes between FQs. For each *in silico* granuloma we calculate: (1) Bacterial load after treatment; (2) Time to sterilization; (3) Treatment failure (% granulomas not sterilized); (4) *In silico* early bactericidal activity (EBA, fold change in simulated bacterial load between day 0 and 2 or day 2 and 7 of treatment); and (5) Efficacy in simulated non-compliance scenario.

As a collective metric of immune response we define an immune score: (Activated macrophages* + Activated IFN-γ-producing T cells* + Activated cytotoxic T cells* + concentration of free TNF-α *)/4; where ‘*’ indicates that the value is normalized to its value at the start of treatment. Similarly, as a collective metric of infection severity we define an infection score: (Infected macrophages * + Chronically Infected macrophages * + Extracellular Replicating Mtb* + Extracellular Nonreplicating Mtb*)/4. Numbers of immune cells and bacteria are counted throughout the entire simulation grid. The concentration of free TNF-α is determined by averaging over the simulation grid compartments inside the granuloma.

## Supporting information

S1 Fig Pharmacodynamic parameter estimation. (PDF)

S2 Fig Fluoroquinolone dynamics in uninvolved rabbit lung samples. (PDF)

S3 Fig Spatial fluoroquinolone distribution in granulomas with rabbit and human PK. (PDF)

S4 Fig Bacterial and immune responses following treatment interruption after 70 days. (PDF)

S1 Text Tissue PK parameter fitting. (PDF)

S1 Table Host immune parameters varied to capture granuloma variability for tissue PK calibration. (PDF)

S2 Table Sensitivity analysis parameter ranges. (PDF)

S3 Table Sensitivity analysis results. (PDF)
