# Supplementary material for: Comparing efficacies of moxifloxacin, levofloxacin and gatifloxacin in tuberculosis granulomas using a multi-scale systems pharmacology approach
Source: PLoS Comput Biol. 2017 Aug 17;13(8):e1005650. doi: 10.1371/journal.pcbi.1005650 (PMC5560534; doi:10.1371/journal.pcbi.1005650)
Supplement: S1 Fig — (PDF) [file pcbi.1005650.s001.pdf]

Pharmacodynamic parameter estimation

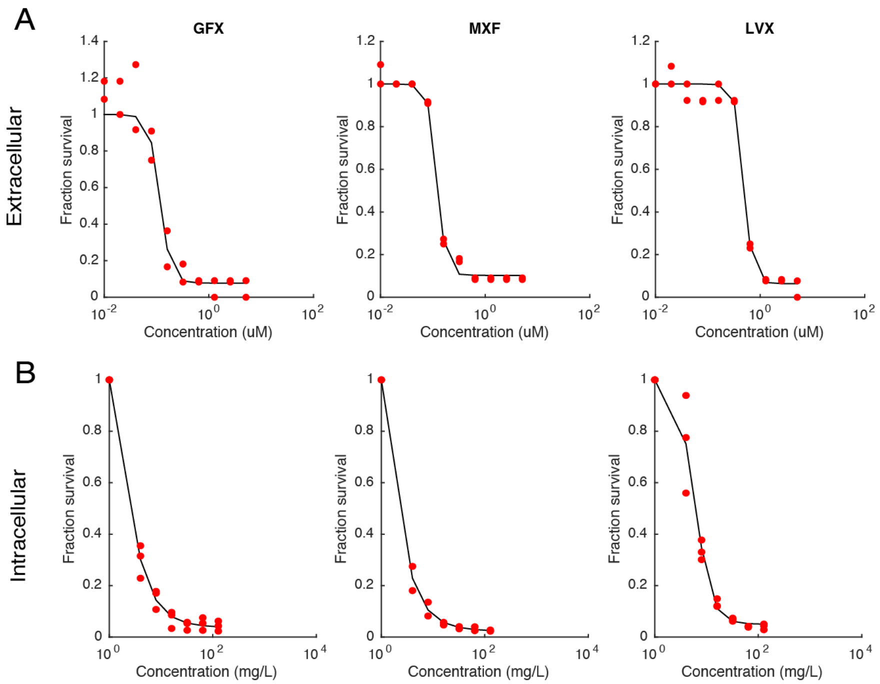

Figure S1: Overlay of in vitro and fitted in silico dose response curves for Mtb in liquid culture after 5 days (A) and in mouse macrophages after 3 days (B). Red data points show in vitro data, and black lines show model fit. X-axis shows antibiotic concentration added in the culture medium.
