# Supplementary material for: Comparing efficacies of moxifloxacin, levofloxacin and gatifloxacin in tuberculosis granulomas using a multi-scale systems pharmacology approach
Source: PLoS Comput Biol. 2017 Aug 17;13(8):e1005650. doi: 10.1371/journal.pcbi.1005650 (PMC5560534; doi:10.1371/journal.pcbi.1005650)
Supplement: S2 Fig — (PDF) [file pcbi.1005650.s002.pdf]

Fluoroquinolone dynamics in uninvolved rabbit lung samples

There are experimental challenges to isolating truly uninvolved lung tissue in Mtb-infected rabbits. Most lung tissue samples showed some inflammation even in the absence of granulomas. Therefore LCMS measurements in uninvolved lung were not used for calibration of tissue PK parameters. Nonetheless, our in silico predicted concentrations of uninfected simulations are in agreement with measurements in uninvolved (i.e. non-granulomatous) rabbit lung samples (Figure S2). Differences between simulation predictions and rabbit data are most notable for MXF and LVX, and involve earlier peak concentrations predicted in the simulations compared to rabbit data.

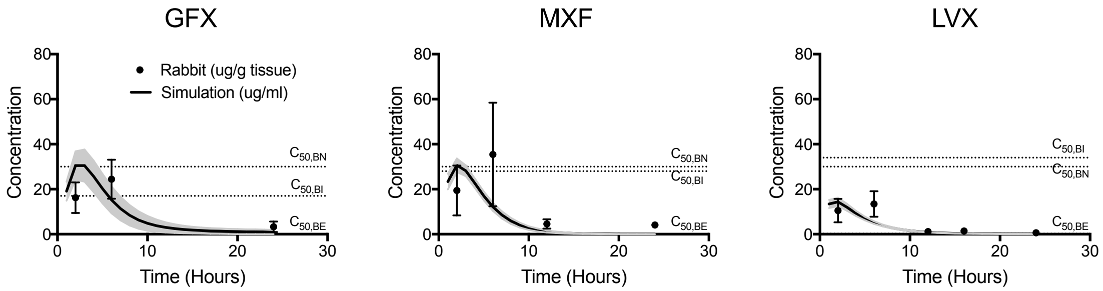

Figure S2: Comparison of average concentrations from simulated uninvolved lung (solid lines) and LCMS measurements in rabbit granulomas (data points). Lines and data points show means and standard deviations for 100 simulations, and between 1 and 67 rabbit samples. Horizontal dashed lines show C_{50} values for intracellular ($C_{50,BI}$), extracellular replicating ($C_{50,BE}$) and extracellular non-replicating bacteria ($C_{50,BN}$).
