# Supplementary material for: Comparing efficacies of moxifloxacin, levofloxacin and gatifloxacin in tuberculosis granulomas using a multi-scale systems pharmacology approach
Source: PLoS Comput Biol. 2017 Aug 17;13(8):e1005650. doi: 10.1371/journal.pcbi.1005650 (PMC5560534; doi:10.1371/journal.pcbi.1005650)
Supplement: S3 Fig — (PDF) [file pcbi.1005650.s003.pdf]

Spatial fluoroquinolone distribution in granulomas with rabbit and human PK

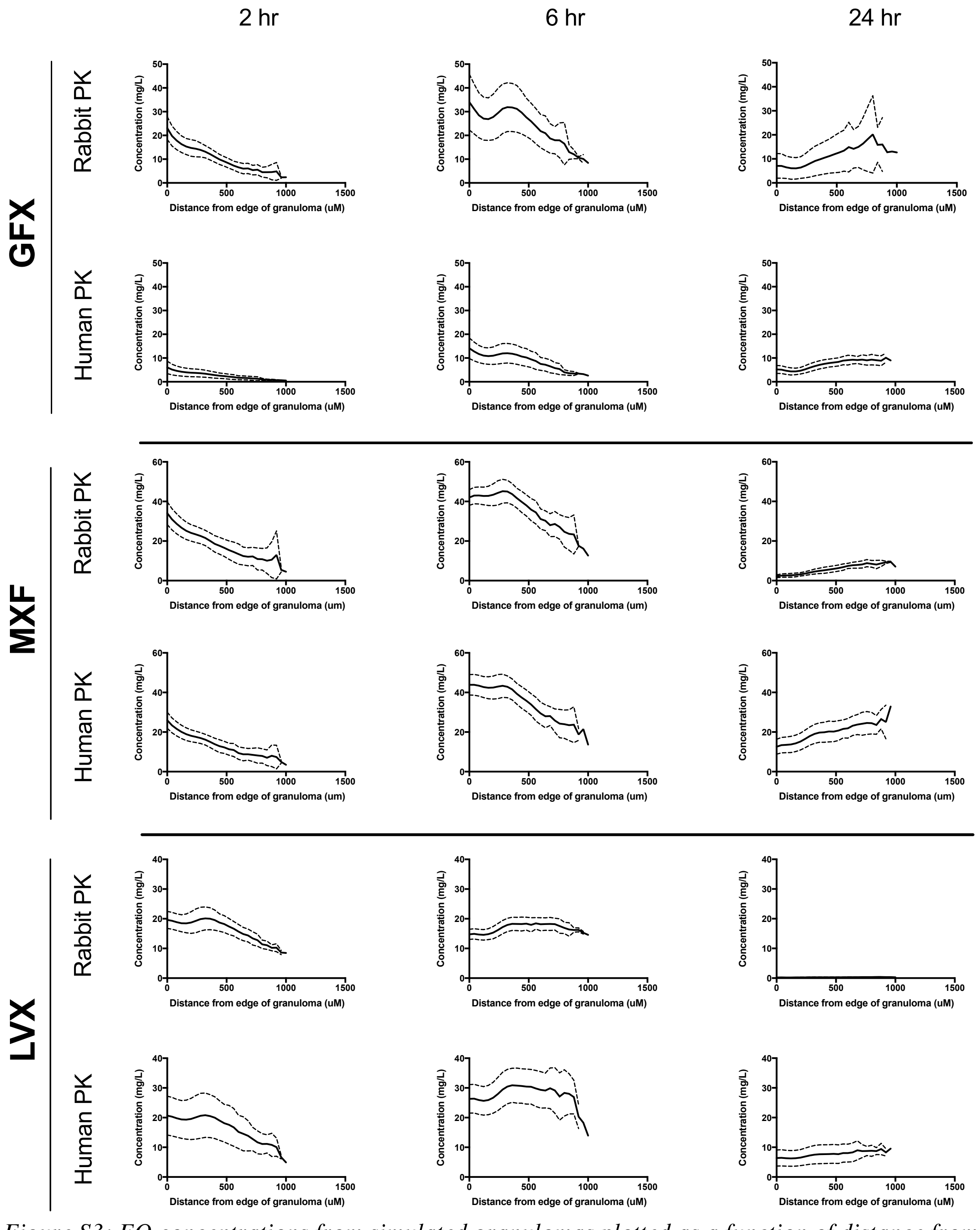

Figure S3: FQ concentrations from simulated granulomas plotted as a function of distance from the edge of the granuloma at 2, 6 and 24 hrs post dose. Solid lines show mean and dashed lines show standard deviation for 100 simulated granulomas.
