# Supplementary material for: Comparing efficacies of moxifloxacin, levofloxacin and gatifloxacin in tuberculosis granulomas using a multi-scale systems pharmacology approach
Source: PLoS Comput Biol. 2017 Aug 17;13(8):e1005650. doi: 10.1371/journal.pcbi.1005650 (PMC5560534; doi:10.1371/journal.pcbi.1005650)
Supplement: S4 Fig — (PDF) [file pcbi.1005650.s004.pdf]

Bacterial and immune responses following treatment interruption after 70 days

Following treatment interruption after 10 days of treatment (at day 390), bacterial load rebounds more quickly and more steeply for GFX and LVX compared to MXF (Figure 10, main text). However, if treatment is interrupted after 70 days (at day 450) the outcomes are more similar between FQs (Figure S4 A-D). This indicates that bacterial and immunological differences between MXF and the other two FQs that are influential during the first 10 days of treatment, have largely been removed at this later time point (Figure S4 E-F)

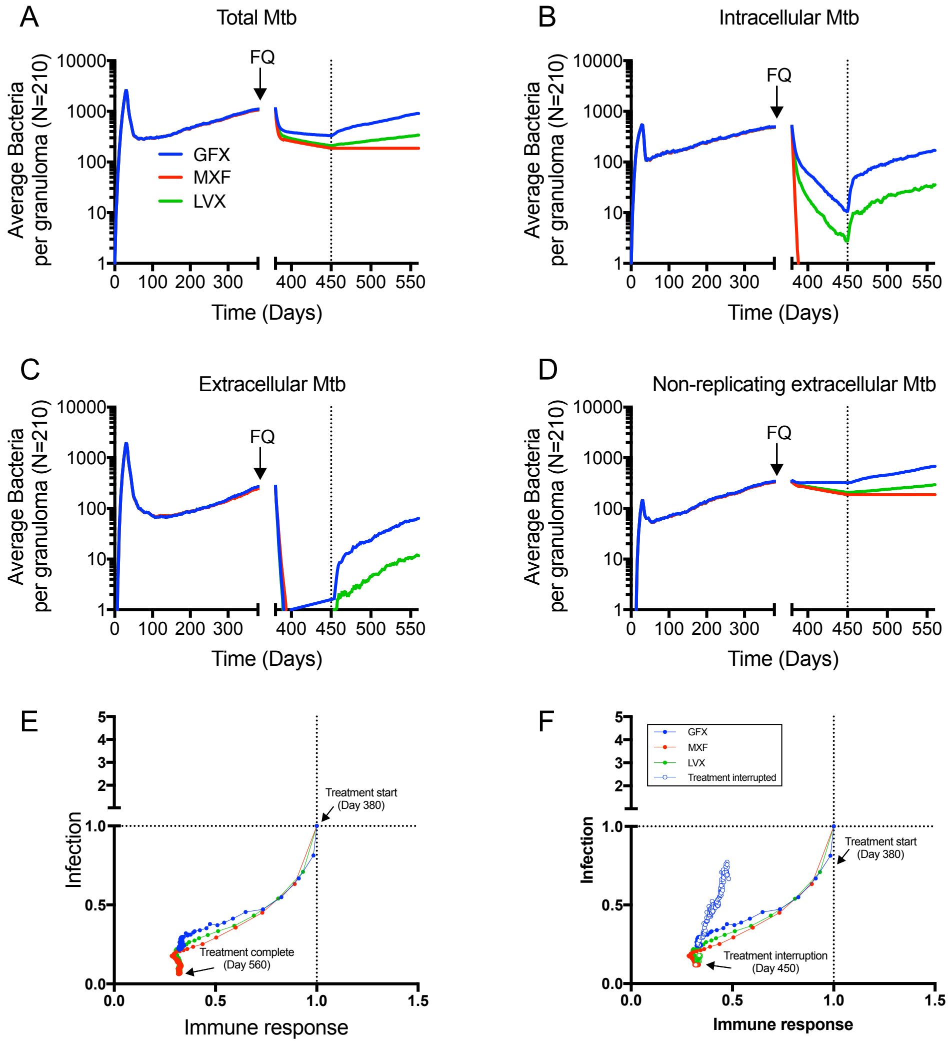

Figure S4: (A-D) Simulations showing intracellular and extracellular bacteria increasing slightly following GFX and LVX interruption, where these populations are eliminated by MXF before day 450. The non-replicating bacterial population shows little change following interruption of any of the FQs. Lines show means of 210 *in silico* granulomas, with infection starting at day 0, daily FQ treatment starting at day 380 (arrows). Treatment is interrupted after 70 days (vertical dotted lines), and the simulation is continued to day 560 without antibiotics. (E-F) Progression of treatment as a function of the collective immune response metric (x-axes) and the collective infection metric (y-axes), during complete treatment (E) and interrupted treatment (F). The start of treatment is located at the intersection of the dotted lines. Filled circles indicate the treatment phase, and open circles indicated progression following treatment interruption.
