# Supplementary material for: Comparing efficacies of moxifloxacin, levofloxacin and gatifloxacin in tuberculosis granulomas using a multi-scale systems pharmacology approach
Source: PLoS Comput Biol. 2017 Aug 17;13(8):e1005650. doi: 10.1371/journal.pcbi.1005650 (PMC5560534; doi:10.1371/journal.pcbi.1005650)
Supplement: S1 Text — (PDF) [file pcbi.1005650.s005.pdf]

Tissue PK parameter fitting

We estimate tissue PK parameters by calibrating *GranSim* to the *in vivo* data summarized in Table 2 and Table 6 in the main text. We use Latin Hypercube sampling to sample the parameter space (See Methods – Sensitivity analysis). The ranges sampled for each parameter are based on a collection of *in vitro* and literature data (see Table below).

FQ diffusivity ranges in tissue are estimated based on molecular weight, logP and the number of hydrogen donor and acceptor sites based on diffusion studies in tumors [1]. Vascular permeability estimates based on molecular radius alone predict vascular permeability of ~5x10^{-5} cm/s for all three FQs [2]. However, we use vascular permeability ranges one log lower than this estimate (5x10^{-6} cm/s), since we noted that the predicted diffusivity dropped by ~10-fold if one includes the physicochemical properties listed above in addition to the molecular size alone. Furthermore, *in vitro* permeability studies showed that MXF has consistently higher permeability than GFX and LVX by 2- to 10-fold [3]. We estimate initial MXF permeability ranges 2-fold higher than GFX and LVX. Cellular uptake ratio estimates are based on *in vitro* cell uptake assays in THP-1 cells described below. Permeability coefficient estimates are based on plasma protein binding measurements [3]. Caseum unbound fractions are estimated from *in vitro* rapid equilibrium dialysis assays described below.

Table: Parameter ranges used for Tissue PK Parameter fitting. The ranges explored during calibration were chosen based on best estimates from experiments and literature for all three FQs. Where no references are given, ranges are based on in vitro data obtained in this work. Final parameter estimates resulting from calibration are given in Table M2 in the main text.

Parameter	Units	MXF		GFX		LVX	
		Experimental/ Literature estimate	Range used in calibration	Experimenta l/Literature estimate	Range used in calibration	Experiment al/Literatur e estimate	Range used in calibration
Effective diffusivity (*D*)	cm^2/s	2.6x10^{-7} [1]	2.6x10^{-8} – 2.6x10^{-6}	7x10^{-7} [1]	7x10^{-8} – 7x10^{-6}	4x10^{-7} [1]	4x10^{-8} – 4x10^{-6}
Cellular accumulation ratio [(2)] (*a*)	-	4.35	0.4 – 40	2.78	0.2 – 20	2.09	0.2 – 20
Vascular permeability (*p*)	cm/s	1x10^{-5} [2]	1x10^{-6} – 1x10^{-4}	5x10^{-6} [2]	5x10^{-7} – 5x10^{-5}	5x10^{-6} [2]	5x10^{-7} – 5x10^{-5}
Permeability coefficient (*PC*)	-	0.5 [3]	0.05 – 5	0.8 [3]	0.08 – 8	0.7 [3]	0.07 – 7
Caseum unbound fraction (f_u)	-	0.13	0.05 – 0.3	0.16	0.05 – 0.3	0.18	0.01 – 0.4
Caseum binding rate constant (k_{fc})	cu^{-1}s^{-1}		0.0002 – 0.2		0.0002 – 0.2		0.0002 – 0.2
Epithelium binding association constant (K_a)	-		0.01 – 0.02		0.01 – 0.02		0.01 – 0.02
Epithelium binding rate constant (k_{fe})	s^{-1}		0.004 – 0.0099		0.004 – 0.0099		0.004 – 0.0099
Cellular exit rate constant (k_{out})	s^{-1}		0.1 – 0.5		0.1 – 0.5		0.1 – 0.5

References

1 Pruijn, F. B., Patel, K., Hay, M. P., Wilson, W. R. & Hicks, K. O. Prediction of Tumour Tissue Diffusion Coefficients of Hypoxia-Activated Prodrugs from Physicochemical Parameters. *Australian Journal of Chemistry* **61**, 687-693 (2008).

2 Schmidt, M. M. & Wittrup, K. D. A modeling analysis of the effects of molecular size and binding affinity on tumor targeting. *Molecular cancer therapeutics* **8**, 2861-2871, doi:10.1158/1535-7163.MCT-09-0195 (2009).

3 Lakshminarayana, S. B. *et al*. Comprehensive physicochemical, pharmacokinetic and activity profiling of anti-TB agents. *The Journal of antimicrobial chemotherapy* **70**, 857-867, doi:10.1093/jac/dku457 (2015).
