# Supplementary material for: Comparing efficacies of moxifloxacin, levofloxacin and gatifloxacin in tuberculosis granulomas using a multi-scale systems pharmacology approach
Source: PLoS Comput Biol. 2017 Aug 17;13(8):e1005650. doi: 10.1371/journal.pcbi.1005650 (PMC5560534; doi:10.1371/journal.pcbi.1005650)
Supplement: S1 Table — (PDF) [file pcbi.1005650.s006.pdf]

Host immune parameters varied to capture granuloma variability for tissue PK calibration

Table S1: Host immune parameter ranges used to generate collections of test granulomas to calibrate tissue PK parameters [1,2]

Host Immune Parameters	Unit*	Range (min – max)
Time to heal caseation	Days	8 – 12
TNF threshold for causing apoptosis	Molecules	900 – 1400
Rate of TNF induced apoptosis	s^{-1}	$1.3x10^{-6} – 2x10^{-6}$
Minimum chemokine concentration allowing chemotaxis	Molecules	0.4 – 0.6
Maximum chemokine concentration allowing chemotaxis	Molecules	380 – 570
Initial macrophage density	Fraction of grid comp.	0.03 – 0.05
Time steps before a resting macrophage can move	Timesteps	2 – 4
Time steps before an activated macrophage can move	Timesteps	15 – 24
Time steps before an infected macrophage can move	Timesteps	135 – 200
TNF threshold for activating NFkB	Molecules	60 – 90
Rate of TNF induced NFkB activation	s^{-1}	$8x10^{-6} – 1.5x10^{-5}$
Probability of resting macrophage killing bacteria		0.1 – 0.15
Adjustment for killing probability of resting macrophages with NFkB activated		0.15 – 0.25
Number of extracellular bacteria in the Moore neighborhood that can activate NFkB	Bacteria	200 – 300
Threshold for intracellular bacteria causing chronically infected macrophages	Bacteria	10 – 15
Threshold for intracellular bacteria causing macrophage to burst	Bacteria	18 – 30
Number of bacteria activated macrophage can phagocytose	Bacteria	4 – 6
Probability of an activated macrophage healing a caseated compartment in its Moore neighborhood		0.004 – 0.007
Number of host cell deaths causing caseation		4 (Caseous granulomas)<br>15 (Cellular granulomas)
Probability of a T-cell moving to the same compartment as a macrophage		0.035 – 0.055
IFN γ –producing T-cell probability of inducing Fas/FasL mediated apoptosis		0.03 – 0.04
IFN γ –producing T-cell probability of producing TNF		0.04 – 0.05
IFN γ –producing T-cell probability of producing IFN		0.3 – 0.45
Cytotoxic T-cell probability of killing a macrophage		0.007 – 0.01
Cytotoxic T-cell probability of, when it kills a macrophage, also killing all of its intracellular bacteria		0.6 – 0.9
Cytotoxic T-cell probability of producing TNF		0.04 – 0.06
Regulatory T-cell probability of deactivating activated macrophage		0.006 – 0.01
Time before maximum recruitment rates are reached	Timesteps*	790 – 1180
Macrophage maximal recruitment probability		0.25 – 0.4
Macrophage chemokine recruitment threshold	Molecules	0.7 – 1
Macrophage TNF recruitment threshold	Molecules	0.009 – 0.015
Macrophage half sat for TNF recruitment	Molecules	1.3 – 2
Macrophage half sat for chemokine recruitment	Molecules	1.8 – 2.6
IFN γ –producing T-cell maximal recruitment probability		0.12 – 0.18
IFN γ –producing T-cell chemokine recruitment threshold	Molecules	0.0.06 – 0.09
IFN γ –producing T-cell TNF recruitment threshold	Molecules	1 – 1.6
IFN γ –producing T-cell half sat for TNF recruitment	Molecules	1 – 1.6

IFN γ –producing T-cell half sat for chemokine recruitment	Molecules	1.5 – 2.5
Cytotoxic T-cell maximal recruitment probability		0.1 – 0.15
Cytotoxic T-cell chemokine recruitment threshold	Molecules	3.6 – 5.4
Cytotoxic T-cell TNF recruitment threshold	Molecules	1 – 1.5
Cytotoxic T-cell half sat for TNF recruitment	Molecules	1 – 1.5
Cytotoxic T-cell half sat for chemokine recruitment	Molecules	7 – 10
Regulatory T-cell maximal recruitment probability		0.02 – 0.04
Regulatory T-cell chemokine recruitment threshold	Molecules	1.5 – 2.5
Regulatory T-cell TNF recruitment threshold	Molecules	1.3 – 2
Regulatory T-cell half sat for TNF recruitment	Molecules	1.8 – 2.7
Regulatory T-cell half sat for chemokine recruitment	Molecules	1.2 – 1.8

*Conversion factor: 10 min/timestep.

References

1 Pienaar, E. *et al*. A computational tool integrating host immunity with antibiotic dynamics to study tuberculosis treatment. *J Theor Biol* **367**, 166-179, doi:10.1016/j.jtbi.2014.11.021 (2015).

2 Pienaar, E., Dartois, V., Linderman, J. J. & Kirschner, D. In silico evaluation and exploration of antibiotic tuberculosis treatment regimens. *BMC systems biology* **9**, 79, doi:10.1186/s12918-015-0221-8 (2015).
