# Supplementary material for: Comparing efficacies of moxifloxacin, levofloxacin and gatifloxacin in tuberculosis granulomas using a multi-scale systems pharmacology approach
Source: PLoS Comput Biol. 2017 Aug 17;13(8):e1005650. doi: 10.1371/journal.pcbi.1005650 (PMC5560534; doi:10.1371/journal.pcbi.1005650)
Supplement: S2 Table — (PDF) [file pcbi.1005650.s007.pdf]

Sensitivity analysis – Parameter ranges

For sensitivity analysis using Partial rank correlations coefficients (PRCC, see Methods), parameters were sampled simultaneously and uniformly in the ranges given in Table S2 using Latin Hypercube sampling (LHS). Significant correlations are summarized in Table S3.

Table S2: Parameters and ranges used in PRCC calculation for sensitivity analysis.

Parameter	Units	Min	Max
**Plasma PK parameters** [2,3]			
Absorption rate constant (k_a)	h^{-1}	1	10
Intercompartmental clearance rate constant (Q)	L/h/kg	1	10
Plasma volume of distribution (V_p)	L/kg	0.1	1
Peripheral volume of distribution (V_{pe})	L/kg	0.1	1
Plasma clearance rate constant (CL)	L/h/kg	0.1	1
**Lung tissue PK parameters** [4]			
Effective diffusivity (D)	cm^2/s	$1x10^{-07}$	$1x10^{-06}$
Cellular accumulation ratio [2] (a)	-	1	10
Vascular permeability (p)	cm/s	$1x10^{-06}$	$1x10^{-05}$
Permeability coefficient (PC)	-	1	10
Caseum unbound fraction (f_u)	-	0.1	0.9
Caseum binding rate constant (k_{fc})	$cu^{-1}s^{-1}$	0.005	0.05
Epithelium binding association constant (K_a)	-	0.01	0.03
Epithelium binding rate constant (k_{fe})	s^{-1}	0.002	0.009
Cellular exit rate constant (k_{out})	s^{-1}	0.02	0.2
**PD parameters** [5]			
Max activity extracellular ($E_{max,BE}$)	s^{-1}	0.001	0.01
Max activity intracellular ($E_{max,BI}$)	s^{-1}	0.001	0.01
C50 for extracellular replicating Mtb ($C_{50,BE}$)	mg/L	0.01	0.1
C50 for extracellular non-replicating Mtb ($C_{50,BN}$) [6]	mg/L	5	50
C50 for intracellular Mtb ($C_{50,BI}$)	mg/L	5	50
Hill constant for intracellular Mtb (H_{BI})	-	1	5
Hill constant for extracellular replicating Mtb (H_{BE})	-	1	5
Hill constant for extracellular non-replicating Mtb (H_{BN})	-	1	5
