# Supplementary material for: Comparing efficacies of moxifloxacin, levofloxacin and gatifloxacin in tuberculosis granulomas using a multi-scale systems pharmacology approach
Source: PLoS Comput Biol. 2017 Aug 17;13(8):e1005650. doi: 10.1371/journal.pcbi.1005650 (PMC5560534; doi:10.1371/journal.pcbi.1005650)
Supplement: S3 Table — (PDF) [file pcbi.1005650.s008.pdf]

Sensitivity analysis - Results

For sensitivity analysis using Partial rank correlations coefficients (PRCC, see Methods), parameters were sampled simultaneously and uniformly in the ranges given in Table S2 using Latin Hypercube sampling (LHS). Significant correlations are summarized in Table S3.

*Table S3: Significant correlations between model parameters and model outputs. Only significantly correlated parameters are shown. Relationship: '+': positive correlation; '-': negative correlation. Significance: *: p < 0.01; **: p < 0.001; ***: p < 0.0001.*

Model outputs	Significant Parameters											
	ka	Vp	CL	a	p	PC	Emax,BI	Emax,BE	C50,BN	C50,BI	HBE	fu
Resting macrophages			+++	---		---	---	+		+++		
Infected macrophages			+++	---		---	---			+++		
Chronically infected macrophages			+++	---		---	---			+++		
Activated macrophages			+++	---		---	---			+++		
IFNg producing T cells			+++	---		---	---			+++		
Activated IFNg producing T cells			+++	---		---	---			+++		
Cytotoxic T cells			+++	---		---	---			+++		
Activated cytotoxic T cells			+++	---		---	---			+++		
Regulatory T cells			+++	---		---	---			+++		
Activated regulatory T cells			+++	---		---	---			+++		
Intracellular Mtb			+++	---		---	---			+++		
Extracellular Mtb			+++	---		---	---	---	+++	+++	+++	
Replicating extracellular Mtb		-	+++	---	+++	---	---	---		+++		+
Non-replicating extracellular Mtb			+++	---		---	---	---	+++	+++	+++	
Total MTb			+++	---		---	---	-	+++	+++	+++	
AUC in Blood (0-24hr)	---	+++	---									
AUC in Granuloma (0-24hr)			---	+++		+++		-				---
TNF			+++	---		---	---			+++		
IL10			+++	---		---	---			+++		
Lesion Size			+++	---		---	---			+++		
Caseation level			+++	---		---	---			+++		
